# Bis(trifluoromethyl)disulfide and the EtP_4_ Phosphazene Base—Formation of a Bench‐Stable [EtP_4_SCF_3_]^+^[SCF_3_]^−^ Salt

**DOI:** 10.1002/chem.70811

**Published:** 2026-02-25

**Authors:** Lukas Hartmann, Katharina Wels, Natalia Tiessen, Beate Neumann, Jan‐Hendrik Lamm, Hans‐Georg Stammler, Berthold Hoge

**Affiliations:** ^1^ Centrum für Molekulare Materialien, Fakultät für Chemie Universität Bielefeld, Universitätsstraße 25 Bielefeld Germany

**Keywords:** disulfide, multinuclear NMR spectroscopy, phosphazene base, single‐crystal X‐ray diffraction, trifluoromethylthiolation

## Abstract

The reaction of the {(Et_2_N)_3_P═N}_3_P═N*^t^* Bu phosphazene base (EtP_4_) with bis(trifluoromethyl)disulfide, (F_3_CS)_2_, selectively affords [EtP_4_SCF_3_][SCF_3_], which arises from formal heterolytic S─S bond cleavage. The phosphazenium salt represents the first structurally characterized SCF_3_ substituted iminium derivative. Subsequent reaction with methyl halides MeX (X = Br, I) leads to selective substitution of the anionic SCF_3_ moiety and facilitates isolation of the corresponding [EtP_4_SCF_3_]X (X = Br, I) salts. Treatment of [EtP_4_SCF_3_][SCF_3_] with trimethylsilyl halides Me_3_SiX (X = Cl, Br) affords halogenophosphonium halide salts [({Et_2_N}_3_P═N)_3_PX]X (X = Cl, Br), via removal of the iminium unit at the phosphazene center. All compounds were characterized by multinuclear NMR spectroscopy, single‐crystal X‐ray diffraction experiments, and elemental analyses. Both SCF_3_ units are chemically addressable and can be employed in further functionalization. In contrast, no reaction of (F_3_CS)_2_ is observed when the iminophosphorane (C_4_H_8_N)_3_P═N*^t^* Bu is employed under comparable conditions, whereas the phosphanes PMe_3_ and P(NEt_2_)_3_ undergo conversion to the corresponding difluorophosphoranes. These observations underline the exceptional ability of the EtP_4_ phosphazene base to promote bond activation and to stabilize the resulting reactive SCF_3_.

## Graphical abstract

The synthesis of a bench‐stable trifluoromethylthiolate‐substituted phosphazenium salt is reported. Reaction of bis(trifluoromethyl)disulfide with the EtP_4_ phosphazene base furnishes this salt in quantitative yield. The compound features a unique trifluoromethylthiolate‐iminium motif and allows for functionalization of both trifluoromethylthiolate moieties.

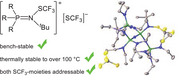

## 1 Introduction

Fluorinated substituents play an important role in modulating the physical, chemical, and biological properties of organic molecules [1, 2]. In recent years, the trifluoromethylthiolate moiety (SCF_3_) has emerged as a particularly attractive structural motif owing to its pronounced lipophilicity, strong electron‐withdrawing properties, and high metabolic stability [3, 4, 5]. These features have prompted the incorporation of the SCF_3_ group into several medicinal and agrochemical products [6, 7, 8, 9, 10]. Consequently, the development of efficient and robust reagents for the introduction of the SCF_3_ unit remains an active area of research.

**FIGURE 1:**
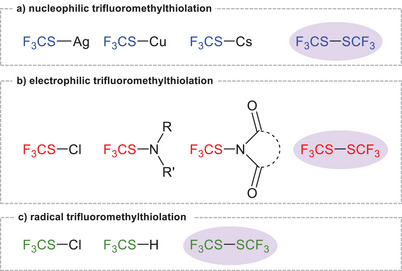
Selected examples for different types of trifluoromethylthiolation reactions.

The nucleophilic trifluoromethylthiolation is a commonly used methodology, facilitated by the convenient availability of suitable substrates. Commonly employed reagents include MSCF_3_ (M = Cs, Ag, Cu, [NMe_4_]) (Figure 1) [4, 11, 12]. For electrophilic trifluoromethylthiolation, reagents such as F_3_CSCl, F_3_CSNRR’ or phthalimide‐based derivatives are used for a variety of transformations [13, 14, 15]. In addition, photochemically generated SCF_3_ radicals further expand the synthetic toolbox for incorporation of the SCF_3_ unit into sp^2^‐ and sp^3^‐hybridized carbon centers [5, 16].

The bis(trifluoromethyl)disulfide ((F_3_CS)_2_) occupies a unique position among SCF_3_ transfer reagents, as it combines the three aforementioned reactivity types. Under appropriate conditions, it can act as an electrophilic trifluoromethylthiolation reagent or serve as a source to generate SCF_3_ radicals [17, 18]. Furthermore, the reduction of (F_3_CS)_2_ with tetrakis(diethylamino)ethylene (TDAE) affords [TDAE]^2+^[SCF_3_]^−^_2_, an efficient and convenient reagent for nucleophilic trifluoromethylthiolation [19]. This breadth of reactivity demonstrates the versatility of (F_3_CS)_2_ as a source of transfer reagents for SCF_3_ groups.

In our previous work, we reported the synthesis of [SeCF_3_] and [TeCF_3_] salts by using the phosphazene base EtP_4_, fluoroform (HCF_3_), and the corresponding elemental chalcogen [20]. This work demonstrates the ability of the phosphazenium cation to stabilize trifluoromethylated chalcogenide anions. However, applying the same conditions on the reaction with elemental sulfur does not lead to the formation of the corresponding [SCF_3_] salt. To overcome this limitation, we turned to (F_3_CS)_2_ as a readily available SCF_3_ source. Recently, a simple preparation of this disulfide using Na[O_2_SCF_3_] and PPh_3_ was reported, providing broad access to this compound [21]. Reaction of (F_3_CS)_2_ with EtP_4_ smoothly affords the [EtP_4_SCF_3_][SCF_3_] salt, representing a rare example in which both SCF_3_ moieties of the disulfide are captured within one molecular entity. Alongside the structural characterization of the salt, its utility as an SCF_3_ transfer reagent was investigated. The products were characterized by multinuclear NMR spectroscopy (Figures S1–S19), single‐crystal X‐ray diffraction, and elemental analysis.

## 2 Results and Discussion

**SCHEME 1:**
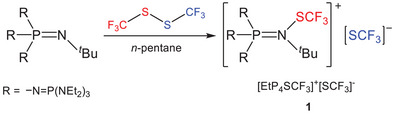
Reaction of the EtP_4_ base with (F_3_CS)_2_ yielding [EtP_4_SCF_3_][SCF_3_] (**1**).

The cleavage of the S─S bond in bis(trifluoromethyl)disulfide by the phosphazene base {(Et_2_N)_3_P═N}_3_P═N*^t^* Bu (EtP_4_) proceeds via formal heterolytic bond scission, affording [EtP_4_SCF_3_][SCF_3_] (Scheme 1). Performing the reaction in *n*‐pentane furnishes the product in quantitative yield as a colorless solid, which is thermally stable up to 104°C.

In its ^31^P NMR spectrum, the central phosphorus atom of **1** appears at −39.3 ppm as a quartet (^2^ *J*_P,P_ = 90 Hz). This resonance is shifted toward the higher field compared to the neutral (−31.3 ppm) and protonated (−33.9 ppm) EtP_4_ base [22]. The three remaining phosphorus atoms in **1** give rise to a quartet of tridecets at 6.0 ppm, exhibiting the same ^2^ *J*_P,P_ coupling constant. In the ^19^F NMR spectrum, the [SCF_3_] anion displays a broad resonance at −7.8 ppm, which is in good agreement with previously reported examples [19, 23]. The SCF_3_ group bound to the phosphazene unit resonates at −48.3 ppm as a doublet (^4^ *J*_F,P_ = 3 Hz) due to coupling to the central phosphorus atom. This coupling pattern is not resolved in the resonance observed in the ^31^P NMR spectrum. Over the course of several days, no signs of decomposition were observed. The molecular composition is confirmed by high‐precision elemental analysis. Crystals, suitable for X‐ray diffraction analysis, are obtained from a concentrated Et_2_O/MeCN solution at −30°C (Table S1).

**FIGURE 2:**
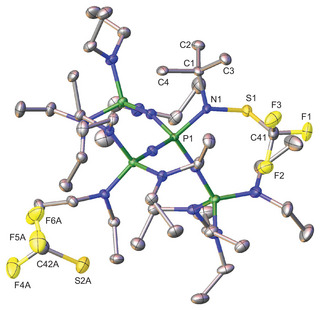
Molecular structure of [EtP_4_SCF_3_][SCF_3_] (**1**). The [SCF_3_] anion is disordered over three positions in a ratio of 72:20:2. Minor occupied, disordered atoms (Figure S29), and hydrogen atoms omitted for clarity. Thermal ellipsoids are shown at the 50% probability level. Selected bond lengths (pm) and angles (°): P1‐N1 174.7(1), N1‐C1 152.7(2), N1‐S1 168.6(1), S1‐C41 181.0(2), C41‐F3 132.9(2), C42A‐S2A 171.5(4), C42A‐F6A 136.2(4), P1‐N1‐S1 116.9(1), P1‐N1‐C1 122.7(1), N1‐S1‐C41 103.5(1), S1‐C41‐F3 115.6(1), S2A‐C42A‐F6A 114.8(3).

Phosphazenium salt **1** (Figure 2) crystallizes in the monoclinic space group *P*2_1_/*c* with four molecules per unit cell. The iminium nitrogen atom N1 adopts an almost planar coordination environment (sum of angles  =  351.18°), consistent with the arrangement observed in [EtP_4_H] salts [22]. The N1‐S1 and S1‐C41 bond lengths of 168.6(1) pm and 181.0(2) pm, respectively, are slightly elongated compared to the corresponding bond distances observed in the neutral SCF_3_ substituted imino compound Cl_6_C_5_NSCF_3_ (166.0 pm and 179.8 pm) [24]. In contrast, the N1‐S1‐C41 angle of 103.5(1)° in **1** is significantly larger than that of the neutral imino analogue (94.90°). Comparison of **1** with SCF_3_ substituted amines reveal only minor variations in bond lengths and angles, suggesting a close structural relation [25, 26, 27].

The [SCF_3_] anion exhibits a shortened S─C bond length (171.5(4) pm) along with elongated C─F bonds (av. 138.3 pm) compared to the SCF_3_ group (181.0(2) pm, av. 133.5 pm) at the phosphazene center, consistent with a partial double bond character in the anionic SCF_3_ moiety. Remaining structural parameters are in line with those reported by the groups of Mews and Röschenthaler [19, 23].

**SCHEME 2:**
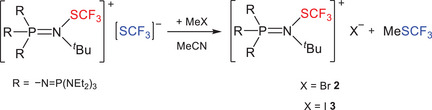
Reaction of [EtP_4_SCF_3_][SCF_3_] (**1**) with MeX (X = Br, I) yields corresponding halide salts and MeSCF_3_.

After establishing convenient access to phosphazenium salt **1**, the reactivity is subsequently investigated. Reaction of **1** with MeX (X = Br, I) furnishes the corresponding phosphazenium halide salts **2** and **3** (Scheme 2). The selective substitution of the [SCF_3_] anion *via* formation of MeSCF_3_ proceeds without affecting the cationic phosphazenium framework.

The ^31^P and ^19^F NMR spectra of the phosphazenium salts **2** and **3** are almost identical to those of **1**. Only the resonance of the [SCF_3_] anion is absent, whereas the doublet caused by the nitrogen‐bonded SCF_3_ moiety remains in the ^19^F NMR spectrum. This observation indicates preservation of the phosphazenium moiety and is consistent with the selective formation of the halide salts. Elemental analysis further confirms this assignment. Crystals of **2** and **3**, suitable for X‐ray diffraction experiments, are obtained from an Et_2_O/CHCl_3_ solution at −30°C (Table S1). Structural parameters of the cations are essentially identical to those observed in the molecular structure of **1** and will not be discussed further (structures depicted in the Supporting Information, Section 6, Figures S31 and S32).

**SCHEME 3:**
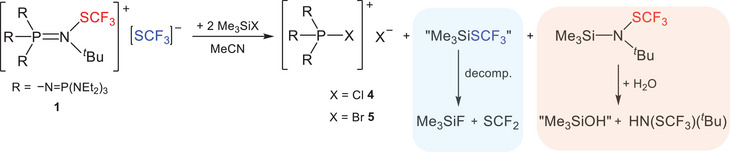
Reaction of [EtP_4_SCF_3_][SCF_3_] (**1**) with Me_3_SiX (X = Cl, Br).

Interestingly, while the reaction of **1** with MeX (X = Br, I) leads to the selective substitution of the anionic SCF_3_ moiety, treatment of **1** with Me_3_SiX (X = Cl, Br) allows both SCF_3_ groups to be addressed. This results in the formation of halogenophosphonium halide salts [({Et_2_N}_3_P═N)_3_PX]X (X = Cl, **4**; X = Br, **5**) (Scheme 3). Whereas chlorophosphonium **4** is isolated, bromophosphonium **5** is not obtained in pure form, due to its pronounced hydrolysis lability. Only NMR spectroscopic data from the reaction mixture and a solid‐state structure are obtained, thus a full characterization is not achieved.

A more detailed understanding of this transformation is obtained by monitoring the reaction of **1** with Me_3_SiCl. The Initial step affords Me_3_SiSCF_3_, which is unstable at ambient temperature, as reported by the group of Clark [28]. Decomposition yields Me_3_SiF and SCF_2_, the latter undergoing oligomerization. In the ^19^F NMR spectrum of the reaction mixture, corresponding resonances are observed at approximately −40 ppm. To yield chlorophosphonium **4**, the formation of Me_3_SiN(SCF_3_)(*^t^* Bu) is required. However, this compound is neither isolated nor detected via GC‐/MS measurements of the reaction mixture. Only a tentative assignment based on the NMR spectroscopic data is made, which exhibits signals in the range expected for the proposed species (for more details, see Supporting Information, Section 4.6, Figure S20).

In the ^31^P NMR spectrum, the central phosphorus atom in chlorophosphonium **4** gives rise to a quartet at −31.6 ppm with a ^2^ *J*_P,P_ coupling constant of 37 Hz, significantly decreased compared to the corresponding coupling constant (^2^ *J*_P,P_ = 90 Hz) in **1**. The remaining phosphorus atoms resonate at 15.6 ppm as a doublet of the tridecets, exhibiting the same ^2^ *J*_P,P_ coupling constant. Elemental analysis confirms the composition of the salt. For bromophosphonium **5**, the central phosphorus atom resonates at −51.6 ppm (^2^ *J*_P,P_ = 34 Hz) as a quartet, shifted by 20 ppm toward a higher field compared to **4**. Other phosphorus atoms give rise to a broad signal at 15.7 ppm. Crystals, suitable for X‐ray diffraction analysis, of **4** ·[H_3_N*^t^* Bu]Cl and **5** ·[H_3_N*^t^* Bu]Br are grown from a MeCN/Et_2_O solution at −30°C (Table S2). The [H_3_N*^t^* Bu]X (X = Cl, Br) presumably originates from the decomposition of HN(SCF_3_)(*^t^* Bu).

**FIGURE 3:**
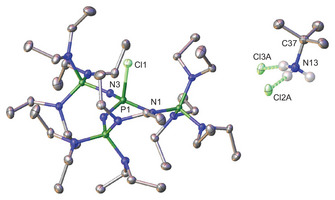
Molecular structure of [({Et_2_N}_3_P=N)_3_PCl]Cl·[H_3_N^t^Bu]Cl. Minor occupied, disordered atoms (Figure S30), and hydrogen atoms (except those attached to N13) are omitted for clarity. Thermal ellipsoids are shown at the 50% probability level. Selected bond lengths(pm) and angles (°): P1‐Cl1 209.8(1), P1‐N1 158.2(1), P1‐N3 158.1(1), N13‐C37 150.7(2), Cl1‐P1‐N1 105.7(1), Cl1‐P1‐N3 105.9(1).

[({Et_2_N}_3_P═N)_3_PCl]Cl · [H_3_N*^t^* Bu]Cl (Figure 3) crystallizes in the triclinic space group *P* $\bar{1}$ with two molecules per unit cell. The central phosphorus atom adopts an almost tetrahedral coordination geometry (τ_4_ = 0.94), similar to that observed in **1** (same τ_4_ value) [29]. The P1‐Cl1 bond length of 209.8(1) pm is comparable to the value reported for a guanidine‐substituted chlorophosphonium compound (208.58(8) pm) by Dielmann and Hahn [30]. In contrast, the chlorophosphonium compound reported by Minkwitz bearing methyl groups exhibits a notably shorter P─Cl bond length (199.0(1) pm) [31].

**FIGURE 4:**
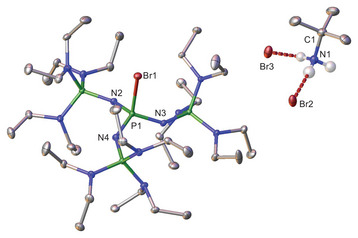
Molecular structure of [({Et_2_N}_3_P=N)_3_PBr]Br·[H_3_N^t^Bu]Br. Minor occupied atoms and hydrogen atoms (except those attached to N1) are omitted for clarity. Thermal ellipsoids are shown at the 50% probability level. Selected bond lengths (pm) and angles (°): P1‐Br1 228.0(1), P1‐N2 157.9(2), P1‐N3 157.8(2), N1‐C1 150.3(3), Br1‐P1‐N2 105.2(1), Br1‐P1‐N3 105.5(1).

[({Et_2_N}_3_P═N)_3_PBr]Br · [H_3_N*^t^* Bu]Br (Figure 4) crystallizes in the triclinic space group *P*‐1 with two molecules per unit cell. Structural parameters surrounding the central phosphorus atom closely resemble those observed in chlorophosphonium **4**. Comparison of the P1─Br1 bond length (228.0(1) pm) with the corresponding distances in [Mes_3_PBr]^+^ (220.3(1) pm) and the azaphosphatrane [(*^i^* BuNCH_2_CH_3_)_3_NPBr]^+^ (227.6(1) pm) reveals similar values [32, 33, 34].

**SCHEME 4:**
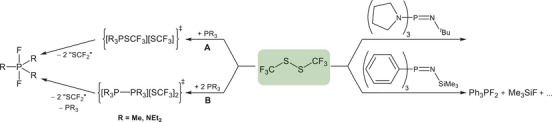
Reaction of (F_3_CS)_2_ with iminophosphoranes ((C_4_H_8_N)_3_P=N^t^Bu and Ph_3_P=NSiMe_3_) and phosphanes (PMe_3_ and P(NEt_2_)_3_).

In an effort to extend this chemistry to related systems and gain access to compounds structurally analogous to **1**, we also investigated the reactivity of (F_3_CS)_2_ with phosphanes and iminophosphoranes, as these substrates are more readily available than the EtP_4_ phosphazene base (Scheme 4).

Under the applied conditions, no reaction is observed between (C_4_H_8_N)_3_P═N*^t^* Bu and (F_3_CS)_2_ according to ^31^P and ^19^F NMR spectroscopic investigations (Figures S21 and S22). In contrast, the reaction with Ph_3_P═NSiMe_3_ results in decomposition, giving Ph_3_PF_2_, Me_3_SiF, and additional unassigned species (Figures S23 and S24). The phosphanes (PMe_3_ and P(NEt_2_)_3_) readily undergo reaction to also yield the corresponding difluorophosphoranes (Figures S25–S28). The course of this transformation remains uncertain, but two mechanistic scenarios appear plausible. One possibility parallels the reactivity observed for EtP_4_, proceeding through initial generation of a transient [R_3_PSCF_3_][SCF_3_] salt (R  = Me, NEt_2_), which subsequently decomposes via SCF_2_ formation (Scheme 4, pathway **A**). An alternative pathway could involve two sequential single‐electron transfers from the phosphanes to (F_3_CS)_2_, ultimately yielding the diphosphonium salt [R_3_PPR_3_][SCF_3_]_2_ (R  =  Me, NEt_2_) (Scheme 4, pathway **B**), which readily decomposes by formation of PR_3_ and SCF_2_. This latter scenario closely resembles the reduction of (F_3_CS)_2_ with TDAE [19]. It should be emphasized that both mechanistic interpretations remain speculative and should be considered with caution. However, in both cases, the proposed intermediary formed salts undergo decomposition, highlighting the unique ability of our employed phosphazenium cation to stabilize such reactive species.

## 3 Conclusion

In this contribution, we explored the reactivity of the phosphazene base EtP_4_ with bis(trifluoromethyl)disulfide, which led to the selective formation of [EtP_4_SCF_3_][SCF_3_]. This transformation corresponds to the formal heterolytic cleavage of the S─S bond and provides access to the first example of a structurally characterized SCF_3_ substituted iminium compound. Subsequent treatment of this species with MeX (X = Br, I) provided access to the corresponding [EtP_4_SCF_3_]X (X = Br, I) salts, while reaction with Me_3_SiX (X = Cl, Br) resulted in removal of the iminium unit off the phosphazenium center and formation of the halogenophosphonium halide salts [({Et_2_N}_3_P═N)_3_PX]X (X = Cl, Br).

In an attempt to prepare structurally related species, (F_3_CS)_2_ was treated with the iminophosphorane (C_4_H_8_N)_3_P═N*^t^* Bu. However, no reaction was observed under the applied conditions, whereas the reaction with Ph_3_P═NSiMe_3_ led to decomposition. The phosphanes PMe_3_ and P(NEt_2_)_3_ readily reacted with the disulfide, yielding the corresponding difluorophosphoranes. Two plausible mechanistic pathways for this transformation were proposed, but both remain speculative and should be regarded with caution.

Nevertheless, the results demonstrate the exceptional versatility of the EtP_4_ phosphazene base, capable of promoting selective bond activation while simultaneously stabilizing highly reactive species. The [EtP_4_SCF_3_][SCF_3_] salt exhibits a remarkable thermal stability with only limited hydrolytic sensitivity, further emphasizing the robustness of this system.

## Conflicts of Interest

The authors declare no conflicts of interest.

## Supporting information




**Supporting File 1**: The authors have cited additional references within the Supporting Information.

## References

[1] J. Wang , M. Sánchez‐Roselló , J. L. Aceña , et al., “Fluorine in Pharmaceutical Industry: Fluorine‐Containing Drugs Introduced to the Market in the Last Decade (2001–2011),” Chemical Reviews 114 (2014): 2432–2506, 10.1021/cr4002879. 24299176

[2] W. Zhang and Y. Liang , “The Wide Presence of Fluorinated Compounds in Common Chemical Products and the Environment: A Review,” Environmental Science and Pollution Research 30 (2023): 108393–108410, 10.1007/s11356-023-30033-6. 37775629

[3] S. Alazet and T. Billard , “Electrophilic Aromatic Trifluoromethylthiolation With the Second Generation of Trifluoromethanesulfenamide,” Synlett 26 (2014): 76–78.

[4] W. Tyrra , D. Naumann , B. Hoge , and Y. L. Yagupolskii , “A New Synthesis of Trifluoromethanethiolates—Characterization and Properties of Tetramethylammonium, Cesium and Di(Benzo‐15‐Crown‐5)Cesium Trifluoromethanethiolates,” Journal of Fluorine Chemistry 119 (2003): 101–107, 10.1016/S0022-1139(02)00276-2.

[5] M. Zhang , J.‐H. Lin , and J.‐C. Xiao , “A Readily Available Trifluoromethylation Reagent and Its Difunctionalization of Alkenes,” Organic Letters 23 (2021): 6079–6083, 10.1021/acs.orglett.1c02146. 34296876

[6] G. W. Counts , D. Gregory , D. Zeleznik , and M. Turck , “Cefazaflur, a New Parenteral Cephalosporin: In Vitro Studies,” Antimicrobial Agents and Chemotherapy 11 (1977): 708–711, 10.1128/AAC.11.4.708. 324399 PMC352055

[7] J. N. Andre , L. G. Dring , G. Gillet , and C. Mas‐Chamberlin , “The Metabolism in Rat of Tiflorex, a m‐Trifluoromethylthio‐Substituted Phenylisopropylamine [Proceedings],” British Journal of Pharmacology 66 (1979): 506. PMC2043666 526774

[8] P. Laczay , G. Vörös , and G. Semjén , “Comparative Studies on the Efficacy of Sulphachlorpyrazine and Toltrazuril for the Treatment of Caecal Coccidiosis in Chickens,” International Journal for Parasitology 25 (1995): 753–756, 10.1016/0020-7519(94)00180-V. 7657461

[9] L. M. Yagupolskii , I. I. Maletina , K. I. Petko , et al., “New Fluorine‐Containing Hypotensive Preparations,” Journal of Fluorine Chemistry 109 (2001): 87–94, 10.1016/S0022-1139(01)00382-7.

[10] R. Islam and J. W. Lynch , “Mechanism of Action of the Insecticides, Lindane and Fipronil, on Glycine Receptor Chloride Channels,” British Journal of Pharmacology 165 (2012): 2707–2720, 10.1111/j.1476-5381.2011.01722.x. 22035056 PMC3423232

[11] G. Teverovskiy , D. S. Surry , and S. L. Buchwald , “Pd‐Catalyzed Synthesis of Ar–SCF_3_ Compounds Under Mild Conditions,” Angewandte Chemie International Edition 50 (2011): 7312–7314, 10.1002/anie.201102543. 21692157 PMC3395331

[12] S. Munavalli , A. Bashir‐Hashemi , D. K. Rohrbaugh , and H. D. Drust , “Trifluoromethylthiocubanes and (Trifluoromethylthio)Cubylcubanes,” Phosphorus, Sulfur, and Silicon and the Related Elements 181 (2006): 435–445, 10.1080/104265091001281.

[13] D. I. Rossman , A. J. Muller , and E. O. Lewis , “Some New Chemistry of Perfluoro‐t‐Butylsilver,” Journal of Fluorine Chemistry 55 (1991): 221–224, 10.1016/S0022-1139(00)80125-6.

[14] F. Baert , J. Colomb , and T. Billard , “Electrophilic Trifluoromethanesulfanylation of Organometallic Species With Trifluoromethanesulfanamides,” Angewandte Chemie International Edition 51 (2012): 10382–10385, 10.1002/anie.201205156. 22976943

[15] T. Bootwicha , X. Liu , R. Pluta , I. Atodiresei , and M. Rueping , “ *N* ‐Trifluormethylthiophthalimid: Ein Stabiles, Elektrophiles SCF_3_ ‐ Reagens und Seine Anwendung in der Katalytischen Asymmetrischen Trifluormethylsulfenylierung,” Angewandte Chemie 125 (2013): 13093–13097, 10.1002/ange.201304957.

[16] M. Li , B. Zhou , X.‐S. Xue , and J.‐P. Cheng , “Establishing the Trifluoromethylthio Radical Donating Abilities of Electrophilic SCF_3_ ‐Transfer Reagents,” Journal of Organic Chemistry 82 (2017): 8697–8702, 10.1021/acs.joc.7b01771. 28737408

[17] R. Boese , A. Haas , M. Lieb , and U. Roeske , “Tris(Perfluororganochalkogenyl)Methyl‐Verbindungen: Synthesen, Strukturen und Eigenschaften,” Chemische Berichte 127 (1994): 449–455, 10.1002/cber.19941270302.

[18] G. Haran and D. W. A. Sharp , “Photochemically Initiated Reactions of Bistrifluoromethyl Disulphide With Olefins,” Journal of the Chemical Society, Perkin Transactions 1 (1972): 34, 10.1039/p19720000034.

[19] A. Kolomeitsev , M. Médebielle , P. Kirsch , E. Lork , and G.‐V. Röschenthaler , “Synthesis, Structure and Reactivity of a Trifluoromethyl Sulfide Anionic Salt Stabilized With Tetrakis(Dimethylamino)Ethylene Dication (TDAE^2+^),” Journal of the Chemical Society, Perkin Transactions 1 (2000): 2183–2185, 10.1039/b002252g.

[20] K. Wels , B. Neumann , H.‐G. Stammler , and B. Hoge , “Preparation and Characterization of Phosphazenium Trifluoromethylselanide and Trifluoromethyltellanide Salts Starting From HCF_3_ ,” Inorganic Chemistry 64 (2025): 13456–13462, 10.1021/acs.inorgchem.5c02006. 40554697

[21] F. Chen , L. Jiang , C. Hu , J. Liu , and W. Yi , “Photocatalyzed Ditrifluoromethylthiolation of alkenes With CF_3_SO_2_Na,” Science China Chemistry 67 (2024): 587–594, 10.1007/s11426-023-1781-6.

[22] R. F. Weitkamp , B. Neumann , H.‐G. Stammler , and B. Hoge , “Generation and Applications of the Hydroxide Trihydrate Anion, [OH(OH_2_)_3_]^−^, Stabilized by a Weakly Coordinating Cation,” Angewandte Chemie International Edition 58 (2019): 14633–14638, 10.1002/anie.201908589. 31373109 PMC6790940

[23] E. Lork , D. Viets , M. Müller , and R. Mews , “Bis(Dimethylamino)Trifluoromethylsulfonium Salze: [CF_3_S(NMe_2_)_2_]^+^[Me_3_SiF_2_]^−^, [CF_3_S(NMe_2_)_2_]^+^[HF_2_]^−^ und [CF_3_S(NMe_2_)_2_]^+^[CF_3_S]^−^ ,” Zeitschrift für anorganische und allgemeine Chemie 630 (2004): 2692–2696, 10.1002/zaac.200400242.

[24] A. H. L. and T. Mischo , “The Preparation of Thiazyltrifluoromethane CF_3_SN, the Hexachloro‐3‐Cyclopentenylidenaminosulfides C_5_Cl_6_NSX (X = CF_3_, Cl, NSO, SCF_3_, C_5_Cl_6_N) and the Crystal Structures of C_5_Cl_6_NSX (X = CF_3_, Cl),” Canadian Journal of Chemistry 67 (1989): 1902–1908.

[25] A. Haas , M. Häberlein , and C. Krüger , “(Trifluormethylthioamino)borane, III. Darstellung und Eigenschaften der [Bis(Trifluormethylthio)Amino]Borane,” Chemische Berichte 109 (1976): 1769–1778, 10.1002/cber.19761090520.

[26] C. Xu , B. Ma , and Q. Shen , “ *N*‐Trifluoromethylthiosaccharin: An Easily Accessible, Shelf‐Stable, Broadly Applicable Trifluoromethylthiolating Reagent,” Angewandte Chemie International Edition 53 (2014): 9316–9320, 10.1002/anie.201403983. 25045031

[27] P. Zhang , M. Li , X.‐S. Xue , et al., “ *N* ‐Trifluoromethylthio‐Dibenzenesulfonimide: A Shelf‐Stable, Broadly Applicable Electrophilic Trifluoromethylthiolating Reagent,” Journal of Organic Chemistry 81 (2016): 7486–7509, 10.1021/acs.joc.6b01178. 27441822

[28] D. J. Adams , S. J. Tavener , and J. H. Clark , “Reactions of Silver(I) Trifluoromethanethiolate With Halotrimethylsilanes: In Situ Generation of Trimethylsilyl Trifluoromethyl Sulfide,” Journal of Fluorine Chemistry 90 (1998): 87–91, 10.1016/S0022-1139(98)00163-8.

[29] L. Yang , D. R. Powell , and R. P. Houser , “Structural Variation in Copper(I) Complexes With Pyridylmethylamide Ligands: Structural Analysis With a New Four‐coordinate Geometry Index, τ_4_ ,” Dalton Transactions (2007): 955–964, 10.1039/B617136B. 17308676

[30] M. D. Böhme , T. Eder , M. B. Röthel , et al., “Synthesis of *N*‐Heterocyclic Carbenes and Their Complexes by Choloronium Ion Abstraction from 2‐Chloroazolium Salts Using Electron‐Rich Phosphines,” Angewandte Chemie, International Edition 61 (2022): e202202190. 35230738 10.1002/anie.202202190 PMC9401039

[31] R. Minkwitz , G. Medger , and H. Preut , “Schwingungsspektroskopische Untersuchungen an Trimethylphosphonium‐Kationen (CH_3_)_3_PX^+^ (X = H, D) und Kristallstrukturen von (CH_3_)_3_PD^+^SbCl_6_ ^−^ und (CH_3_)_3_PCl^+^SbCl_6_ ^−^ ,” Zeitschrift für anorganische und allgemeine Chemie 614 (1992): 102–108, 10.1002/zaac.19926140818.

[32] D. Schneider , O. Schuster , and H. Schmidbaur , “Bromination of (Phosphine)Gold(i) Bromide Complexes: Stoichiometry and Structure of Products,” Dalton Transactions (2005): 1940, 10.1039/b502861b. 15909040

[33] A. D. Matthews , S. Prasad , N. D. Schley , K. J. Donald , and M. W. Johnson , “On Transannulation in Azaphosphatranes: Synthesis and Theoretical Analysis,” Inorganic Chemistry 58 (2019): 15983–15992, 10.1021/acs.inorgchem.9b02467. 31713428

[34] Deposition numbers 2504752 (for 1), 2504753 (for 2), 2504754 (for 3), 2504755 (for 4), and 2504756 (for 5) contain the supplementary crystallographic data for this paper. These data are provided free of charge by the joint Cambridge Crystallographic Data Centre and Fachinformationszentrum Karlsruhe Access Structures service.

