# Supplementary material for: Bis(trifluoromethyl)disulfide and the EtP4 Phosphazene Base—Formation of a Bench‐Stable [EtP4SCF3]+[SCF3]− Salt
Source: Chemistry. 2026 Feb 25;32(18):e70811. doi: 10.1002/chem.70811 (PMC13174922; doi:10.1002/chem.70811)
Supplement: Supplementary file 1 — Supporting File 1: The authors have cited additional references within the Supporting Information. [file CHEM-32-e70811-s001.docx]

**Supporting Information**

Bis(trifluoromethyl)disulfide and the EtP_4_ Phosphazene Base – formation of a bench-stable [EtP_4_SCF_3_]^+^[SCF_3_]^−^ salt

Lukas Hartmann^a^, Katharina Wels^a^, Natalia Tiessen^a^, Beate Neumann^a^, Jan-Hendrik Lamm^a^, Hans-Georg Stammler^a^, Berthold Hoge^a^*

^a^ Centrum für Molekulare Materialien, Fakultät für Chemie, Universität Bielefeld, Universitätsstraße 25, 33615 Bielefeld, Germany

* Email: b.hoge@uni-bielefeld.de

**Table of Contents**

[**1 General** 3](#_Toc218849154)

[**2 Synthetic procedures** 4](#_Toc218849155)

[**2.1** Synthesis of [EtP_4_SCF_3_][SCF_3_] (**1**) 4](#_Toc218849156)

[**2.2** Synthesis of [EtP_4_SCF_3_]Br (**2**) 5](#_Toc218849157)

[**2.3** Synthesis of [EtP_4_SCF_3_]I (**3**) 5](#_Toc218849158)

[**2.4** Synthesis of [({Et_2_N}_3_P=N)_3_PCl]Cl (**4**) 6](#_Toc218849159)

[**2.5** Attempt for the synthesis of [({Et_2_N}_3_P=N)_3_PBr]Br (**5**) 7](#_Toc218849160)

[**3 Reactivity Studies** 8](#_Toc218849161)

[**3.1** Reactions of iminophosphoranes with (F_3_CS)_2_ 8](#_Toc218849162)

[**3.2** Reactions of phosphanes with (F_3_CS)_2_ 8](#_Toc218849163)

[**4 NMR spectra** 10](#_Toc218849164)

[**4.1** NMR spectra of [EtP_4_SCF_3_][SCF_3_] **(1)** 10](#_Toc218849165)

[**4.2** NMR spectra of [EtP_4_SCF_3_]Br (**2**) 13](#_Toc218849166)

[**4.3** NMR spectra of [EtP_4_SCF_3_]I (**3**) 15](#_Toc218849167)

[**4.4** NMR spectra of [({Et_2_N}_3_P=N)_3_PCl]Cl (**4**) 18](#_Toc218849168)

[**4.5** NMR spectrum of [({Et_2_N}_3_P=N)_3_PBr]Br (**5**) 20](#_Toc218849169)

[**4.6** NMR spectrum from the reaction of **1** with Me_3_SiCl 21](#_Toc218849170)

[**4.7** NMR spectra resulting from reactions of (F_3_CS)_2_ with iminophosphoranes 22](#_Toc218849171)

[**4.7.1** With (C_5_H_10_N)_3_P=N*^t^*Bu 22](#_Toc218849172)

[**4.7.2** With Ph_3_P=NSiMe_3_ 23](#_Toc218849173)

[**4.8** NMR spectra resulting from reactions of (F_3_CS)_2_ with phosphanes 24](#_Toc218849174)

[**4.8.1** With PMe_3_ 24](#_Toc218849175)

[**4.8.2** With P(NEt_2_)_3_ 25](#_Toc218849176)

[**5 X-Ray diffraction data** 26](#_Toc218849177)

[**6 Solid-state structures of compounds 2 and 3** 31](#_Toc218849178)

[**7 References** 33](#_Toc218849179)

**1 General**

All chemicals were purchased from commercial sources and used without further purification. Solvents were dried according to standard procedures. Bis(trifluormethyl)disulfide was thankfully received from Prof. J. Grobe. EtP_4_ was prepared as described in the literature.^[1]^ All operations were conducted using a standard high vacuum system. Handling of nonvolatile compounds were performed under normal Schlenk conditions using dry nitrogen as an inert atmosphere. NMR spectra were recorded on a Bruker Avance 500 Neo using the indicated deuterated solvent. For NMR spectroscopic measurements using non-deuterated solvents, capillaries filled with acetone‑d_6_, CFCl_3_ and O=P(OMe)_3_ were used. Positive shifts are downfield from the external standards Si(CH_3_)_4_ (^1^H, ^13^C), CF_3_Cl (^19^F) and H_3_PO_4_ (^31^P). The O1P or O2P specification refers to the spectral center of the corresponding nucleus in the NMR experiment. Elemental analyses were performed with a HEKAtech Euro EA 3000 apparatus or were conducted by the *Mikroanalytisches Laboratorium Kolbe*. Melting points were measured on a Mettler Toledo Mp70 Melting Point System. X-Ray analyses were performed on a Rigaku Supernova diffractometer using Mo-K*α* (*λ* = 0.71073 Å) or Cu-K*α* (*λ* = 1.54184 Å) radiation. Crystals were kept at 100.0(1) K during data collection. Using Olex2, the structure was solved with SHELXT structure solution program using intrinsic phasing and refined with olex2.refine or SHELXL refinement package using Least square minimization. ^[2−4]^

**2 Synthetic procedures**

**2.1** Synthesis of [EtP_4_SCF_3_][SCF_3_] (**1**)

(F_3_CS)_2_ (910 mg, 4.50 mmol) was condensed onto a solution of EtP_4_ (2.74 g, 3.09 mmol) in *n*-pentane. After thawing, a colorless precipitate formed immediately and the suspension was stirred for 16 h. Volatile components were removed *in vacuo* and [EtP_4_SCF_3_][SCF_3_] (**1**, 3.27 g, 3.00 mmol, 97%) was isolated as colorless solid. Crystals, suitable for X-Ray diffraction analysis, were obtained from a Et_2_O/MeCN-solution at −30 °C. Melting Point: 104 °C (decomposition). Elemental analysis: Calcd (%) C(46.35) H(9.17) F(10.47) N(16.73) P(11.38) S(5.89) Found C(46.41) H(9.23) F(10.52) N(16.67) P(11.34) S(5.82).

^1^H NMR (CD_3_CN, 303 K, 500 MHz): 1.1 (t, ^3^*J*_H,H_ = 7 Hz, 54 H, CH_2_C**H_3_**), 1.5 (s, 9 H, C(C**H_3_**)_3_), 3.1 (dm, ^3^*J*_H,P_ = 10 Hz, ^3^*J*_H,H_ = 7 Hz, 36 H, C**H_2_**CH_3_).

^13^C{^1^H} NMR (CD_3_CN, 303 K, 126 MHz): 13.4 (d, ^3^*J*_C,P_ = 3 Hz, 18 C, CH_2_**C**H_3_), 31.2 (s, 3 C, C(**C**H_3_)_3_), 39.8 (d, ^2^*J*_C,P_ = 6 Hz, 18 C, **C**H_2_CH_3_), 63.1 (d, ^2^*J*_C,P_ = 7 Hz , 1 C, **C**(CH_3_)_3_).

^13^C{^19^F}DEPT135 (O2P = −48 ppm) NMR (CD_3_CN, 303 K, 126 MHz): 130.5 (d, ^3^*J*_C,P_ = 2 Hz, NS**C**F_3_).

No signal observed for the carbon atom of the [SCF_3_]-anion.

^19^F NMR (CD_3_CN, 303 K, 470 MHz): −48.3 (d, ^2^*J*_F,P_ = 3 Hz, 3 F, NSC**F_3_**), −7.8 (s (br), 3 F, SC**F_3_**).

^31^P NMR (CD_3_CN, 303 K, 202 MHz): −39.3 (quart, ^2^*J*_P,P_ = 90 Hz, 1 P, ({Et_2_N}_3_PN)_3_**P**), 6.0 (dtridec, ^2^*J*_P,P_ = 90 Hz, ^3^*J*_H,P_ = 10 Hz, 3 P, ({Et_2_N}_3_**P**N)_3_P).

**2.2** Synthesis of [EtP_4_SCF_3_]Br (**2**)

[EtP_4_SCF_3_][SCF_3_] (**1**, 458 mg, 0.42 mmol) was dissolved in MeCN (10 mL) and MeBr (1.00 mmol) was condensed onto it. After stirring for 3 h, all volatile components were removed and [EtP_4_SCF_3_]Br (**2**, 362 mg, 0.34 mmol, 73%) was isolated as a colorless solid. Crystals, suitable for X-Ray diffraction analysis, were obtained from a Et_2_O/CHCl_3_ solution at −30 °C. Elemental analysis: Calcd. (%) C(46.14) H(9.35) N(17.06) S(3.00) Found C(45.99) H(9.53) N(17.09) S(2.79). Melting point: 178-181 °C (decomposition).

^1^H NMR (CD_3_CN, 303 K, 500 MHz): 1.1 (t, ^3^*J*_H,H_ = 7 Hz, 54 H, CH_2_C**H_3_**), 1.5 (s, 9 H, C(C**H_3_**)_3_), 3.1 (dquart, ^3^*J*_H,P_ = 10 Hz, ^3^*J*_H,H_ = 7 Hz, 36 H, C**H_2_**CH_3_).

^13^C{^1^H} NMR (CD_3_CN, 303 K, 126 MHz): 13.4 (d, ^3^*J*_C,P_ = 3 Hz, 18 C, CH_2_**C**H_3_), 31.2 (s, 3 C, C(**C**H_3_)_3_), 39.7 (d, ^2^*J*_C,P_ = 6 Hz, 18 C, **C**H_2_CH_3_), 63.1 (d, ^2^*J*_C,P_ = 7 Hz , 1 C, **C**(CH_3_)_3_).

^13^C{^19^F}DEPT135 (O2P = −48 ppm) NMR (CD_3_CN, 303 K, 126 MHz): 130.5 (d, ^3^*J*_C,P_ = 2 Hz, NS**C**F_3_).

^19^F NMR (CD_3_CN, 303 K, 470 MHz): −48.4 (d, ^2^*J*_F,P_ = 3 Hz, NSC**F_3_**).

^31^P NMR (CD_3_CN, 303 K, 202 MHz): −38.8 (quart, ^2^*J*_P,P_ = 91 Hz, 1 P, ({Et_2_N}_3_PN)_3_**P**), 6.0 (dtridec, ^2^*J*_P,P_ = 90 Hz, ^3^*J*_H,P_ = 10 Hz, 3 P, ({Et_2_N}_3_**P**N)_3_P).

**2.3** Synthesis of [EtP_4_SCF_3_]I (**3**)

[EtP_4_SCF_3_][SCF_3_] (**1**, 372 mg, 0.34 mmol) was dissolved in MeCN (15 mL) and MeI (96 mg, 0.68 mmol) was condensed onto it. After stirring for 1 h, all volatile components were removed and [EtP_4_SCF_3_]I (**3**, 362 mg, 0.34 mmol, quant.) was isolated as a yellow solid. Crystals, suitable for X-Ray diffraction analysis, were obtained from a Et_2_O/CHCl_3_ solution at −30 °C. Elemental analysis: Calcd. (%) C(44.20) H(8.96) N(16.34) S(2.88) Found C(44.60) H(9.18) N(16.12) S(2.64). Melting point: 148 - 150 °C (decomposition).

^1^H NMR (CD_3_CN, 303 K, 500 MHz): 1.1 (t, ^3^*J*_H,H_ = 7 Hz, 54 H, CH_2_C**H_3_**), 1.5 (s, 9 H, C(C**H_3_**)_3_), 3.1 (dquart, ^3^*J*_H,P_ = 10 Hz, ^3^*J*_H,H_ = 7 Hz, 36 H, C**H_2_**CH_3_).

^13^C{^1^H} NMR (CD_3_CN, 303 K, 126 MHz): 13.5 (d, ^3^*J*_C,P_ = 3 Hz, 18 C, CH_2_**C**H_3_), 31.2 (s, 3 C, C(**C**H_3_)_3_), 39.9 (d, ^2^*J*_C,P_ = 6 Hz, 18 C, **C**H_2_CH_3_), 63.1 (d, ^2^*J*_C,P_ = 7 Hz , 1 C, **C**(CH_3_)_3_).

^13^C{^19^F}DEPT135 (O2P = −48 ppm) NMR (CD_3_CN, 303 K, 126 MHz): 130.5 (d, ^3^*J*_C,P_ = 2 Hz, NS**C**F_3_).

^19^F NMR (CD_3_CN, 303 K, 470 MHz): −48.4 (d, ^2^*J*_F,P_ = 3 Hz, NSC**F_3_**).

^31^P NMR (CD_3_CN, 303 K, 202 MHz): −38.8 (quart, ^2^*J*_P,P_ = 91 Hz, 1 P, ({Et_2_N}_3_PN)_3_**P**), 6.0 (dtridec, ^2^*J*_P,P_ = 90 Hz, ^3^*J*_H,P_ = 10 Hz, 3 P, ({Et_2_N}_3_**P**N)_3_P).

**2.4** Synthesis of [({Et_2_N}_3_P=N)_3_PCl]Cl (**4**)

[EtP_4_SCF_3_][SCF_3_] (**1**, 547 mg, 0.50 mmol) was dissolved in acetone (10 mL) and Me_3_SiCl (267 mg, 2.45 mmol) was added. After stirring for 1 h, all volatile components were removed and the resulting solid was dissolved again in acetone (20 mL). After filtration of the orange suspension, the solution was evaporated to dryness, the obtained oil was washed with *n*-pentane (5 x 5 mL) and [({Et_2_N}_3_P=N)_3_PCl]Cl (**4**, 318 mg, 0.36 mmol, 72%) was obtained as a grey to brown solid. Crystals, suitable for X-Ray diffraction analysis, were obtained from a Et_2_O/MeCN solution at −30 °C. Elemental analysis: Calcd. (%) C(48.80) H(10.24) N(18.97) Found C(47.48) H(10.11) N(17.61). Melting point: 112‑120 °C (decomposition).

^1^H NMR (CDCl_3_, 303 K, 500 MHz): 1.1 (t, ^3^*J*_H,H_ = 6 Hz, 54 H, CH_2_C**H_3_**), 3.1 (dquart, ^3^*J*_H,P_ = 16 Hz, ^3^*J*_H,H_ = 7 Hz, 36 H, C**H_2_**CH_3_).

^13^C{^1^H} NMR (CDCl_3_, 303 K, 126 MHz): 13.5 (d, ^3^*J*_C,P_ = 3 Hz, 18 C, CH_2_**C**H_3_), 39.3 (d, ^2^*J*_C,P_ = 4 Hz, 18 C, **C**H_2_CH_3_).

^31^P NMR (CD_3_CN, 303 K, 202 MHz): −31.2 (quart, ^2^*J*_P,P_ = 37 Hz, 1 P, ({Et_2_N}_3_PN)_3_**P**), 15.6 (dtridec, ^2^*J*_P,P_ = 37 Hz, ^3^*J*_H,P_ = 7 Hz, 3 P, ({Et_2_N}_3_**P**N)_3_P).

**2.5** Attempt for the synthesis of [({Et_2_N}_3_P=N)_3_PBr]Br (**5**)

[EtP_4_SCF_3_][SCF_3_] (**1**, 544 mg, 0.50 mmol) was dissolved in MeCN (15 mL) and Me_3_SiBr (778 mg, 5.08 mmol) was added. After stirring for 10 d, Et_2_O was added to the reaction mixture and crystals, suitable for X-Ray diffraction experiments, were obtained.

^31^P NMR (MeCN (with capillary), 303 K, 202 MHz): −51.6 (quart, ^2^*J*_P,P_ = 34 Hz, 1 P, ({Et_2_N}_3_PN)_3_**P**), 15.6 (m (br), 3 P, ({Et_2_N}_3_**P**N)_3_P).

**3 Reactivity Studies**

**3.1** Reactions of iminophosphoranes with (F_3_CS)_2_

(C_5_H_10_N)_3_P=N*^t^*Bu (214 mg, 0.68 mmol) was dissolved in *n*-pentane (15 mL) and (F_3_CS)_2_ (260 mg, 1.29 mmol) was condensed onto the solution. The resulting colorless solution was subjected to ^19^F and ^31^P NMR spectroscopic investigation.

^19^F NMR (*n*-pentane (with capillary), 303 K, 470 MHz): −46.5 (s, (**F_3_**CS)_2_).

^31^P NMR (*n*-pentane (with capillary), 303 K, 202 MHz): 14.0 (s (br), (C_5_H_10_N)_3_**P**=N*^t^*Bu).

Ph_3_P=NSiMe_3_ (333 mg, 0.95 mmol) was suspended in Et_2_O (15 mL) and (F_3_CS)_2_ (262 mg, 1.30 mmol) was condensed onto it. After stirring for 16 h, the volatile components were removed from the yellow suspension. The resulting solid was subjected to ^19^F and ^31^P NMR spectroscopic investigations.

^19^F NMR (MeCN (with capillary), 303 K, 470 MHz): −45.9 (s, (**F_3_**CS)_2_), −37.1 (d, ^1^*J*_F,P_ = 656 Hz, Ph_3_P**F_2_**).

^31^P NMR (MeCN (with capillary), 303 K, 202 MHz): −53.6 (tm, ^1^*J*_F,P_ = 656 Hz, Ph_3_**P**F_2_).

**3.2** Reactions of phosphanes with (F_3_CS)_2_

An excess of (F_3_CS)_2_ was condensed onto a solution of PMe_3_ or P(NEt_2_)_3_ in Et_2_O. Both solutions turned orange after thawing and were subjected to ^19^F and ^31^P NMR spectroscopic investigations.

**With PMe_3­_:**

^19^F NMR (Et_2_O (with capillary), 303 K, 470 MHz): −46.6 (s, (**F_3_**CS)_2_), −4.50 (d, ^1^*J*_F,P_ = 550 Hz, Me_3_P**F_2_**).

^31^P NMR (Et_2_O (with capillary), 303 K, 202 MHz): −16.2 (ttridec, ^1^*J*_F,P_ = 550 Hz, ^2^*J*_H,P_ = 17 Hz, Me_3_**P**F_2_).

**With P(NEt_2_)_3_:**

^19^F NMR (Et_2_O (with capillary), 303 K, 470 MHz): −59.8 (d, ^1^*J*_F,P_ = 698 Hz, (NEt_2_)_3_P**F_2_**), −46.6 (s, (**F_3_**CS)_2_).

^31^P NMR (Et_2_O (with capillary), 303 K, 202 MHz): −58.7 (tm, ^1^*J*_F,P_ = 698 Hz, (NEt_2_)_3_**P**F_2_).

**4 NMR spectra**

**4.1** NMR spectra of [EtP_4_SCF_3_][SCF_3_] **(1)**


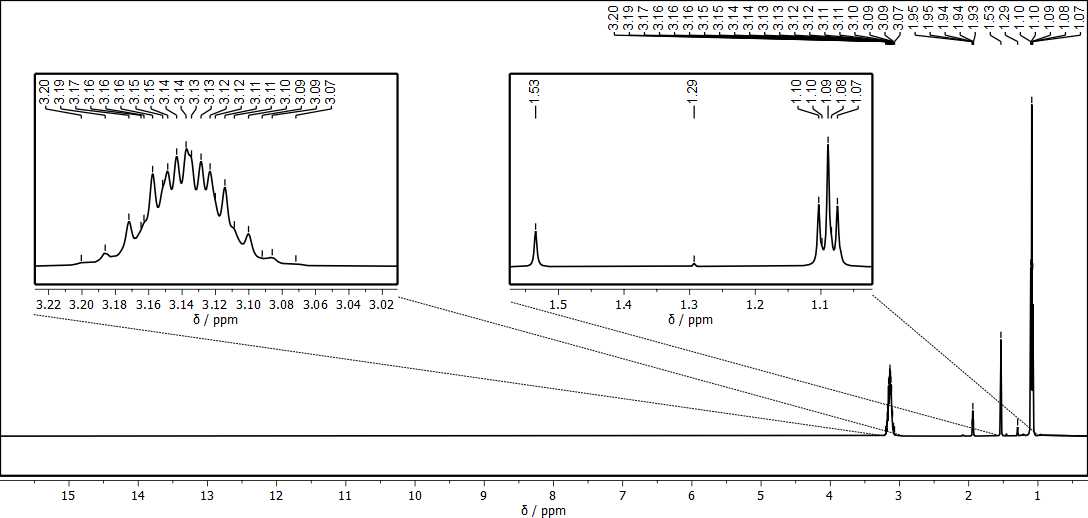


**Figure S1.** ^1^H NMR spectrum of [EtP_4_SCF_3_][SCF_3_] (**1**) in CD_3_CN. Solvent signal: 1.94 ppm.


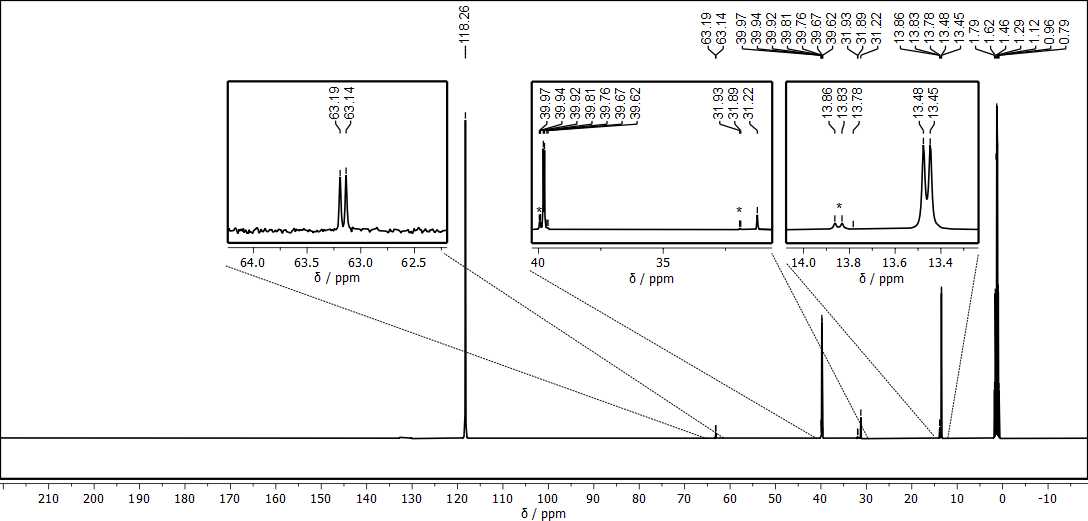


**Figure S2.** ^13^C{^1^H} NMR spectrum of [EtP_4_SCF_3_][SCF_3_] (**1**) in CD_3_CN. Solvent signals: 1.32 ppm and 118.26 ppm. *[EtP_4_H] cation.


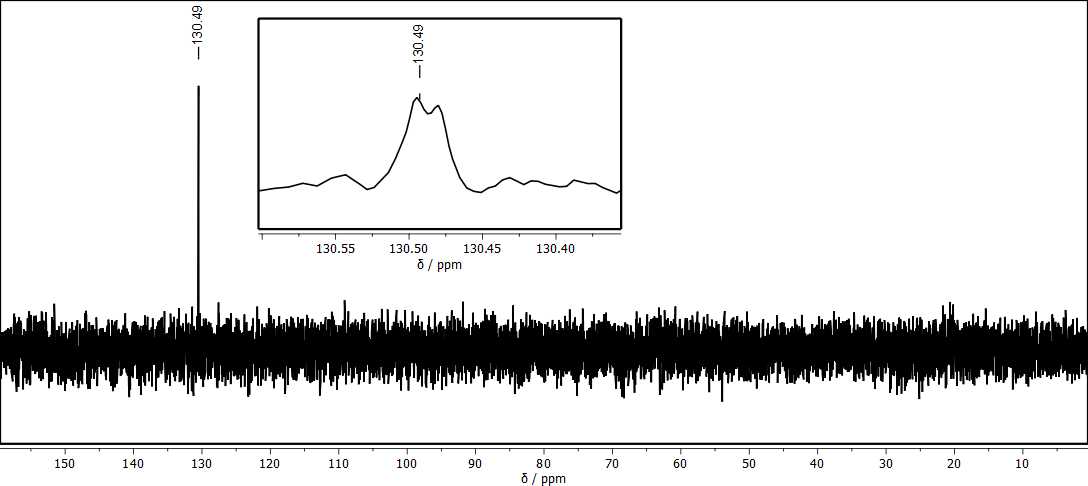


**Figure S3.** ^13^C{^19^F} NMR spectrum of [EtP_4_SCF_3_][SCF_3_] (**1**) in CD_3_CN. O2P = −48.0 ppm.


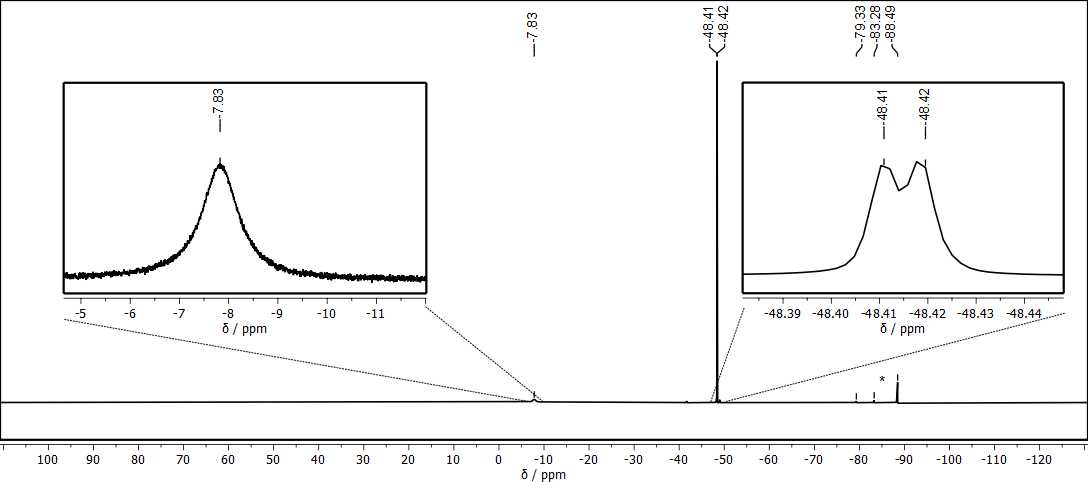


**Figure S4.** ^19^F NMR spectrum of [EtP_4_SCF_3_][SCF_3_] (**1**) in CD_3_CN. O1P = −10.0 ppm. *unidentified impurities.


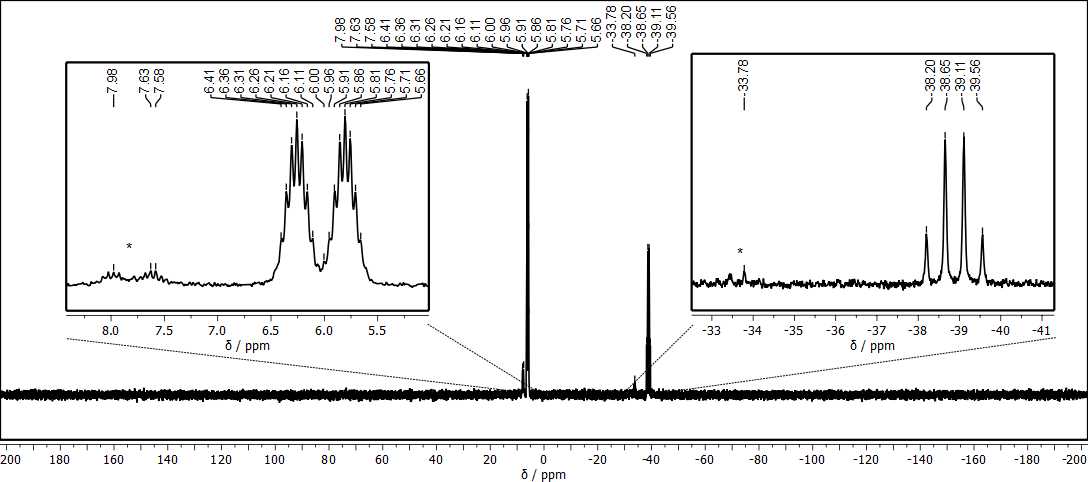


**Figure S5.** ^31^P NMR spectrum of [EtP_4_SCF_3_][SCF_3_] (**1**) in CD_3_CN. *[EtP_4_H] cation.

## **4.2** NMR spectra of [EtP_4_SCF_3_]Br (**2**)


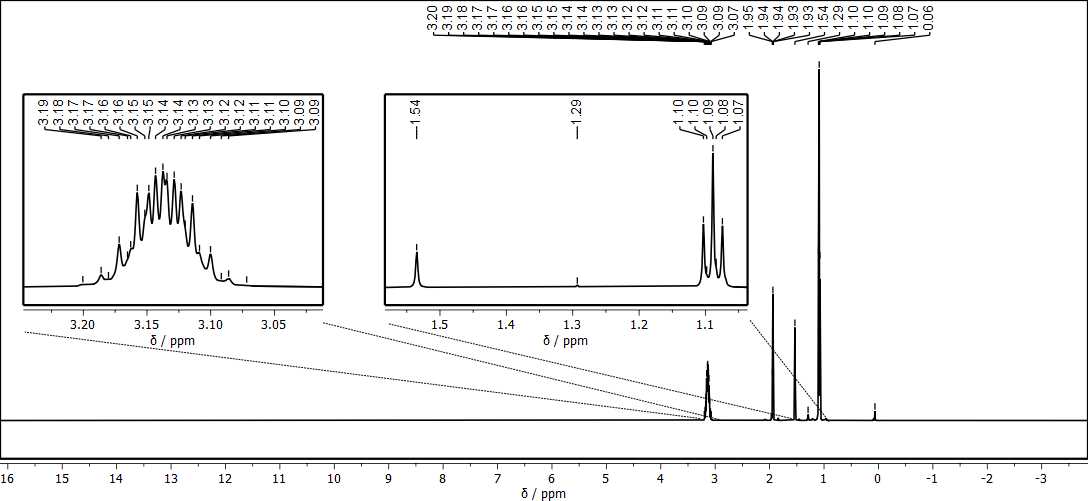


**Figure S6.** ^1^H NMR spectrum of [EtP_4_SCF_3_]Br (**2**) in CD_3_CN. Solvent signal: 1.94 ppm.


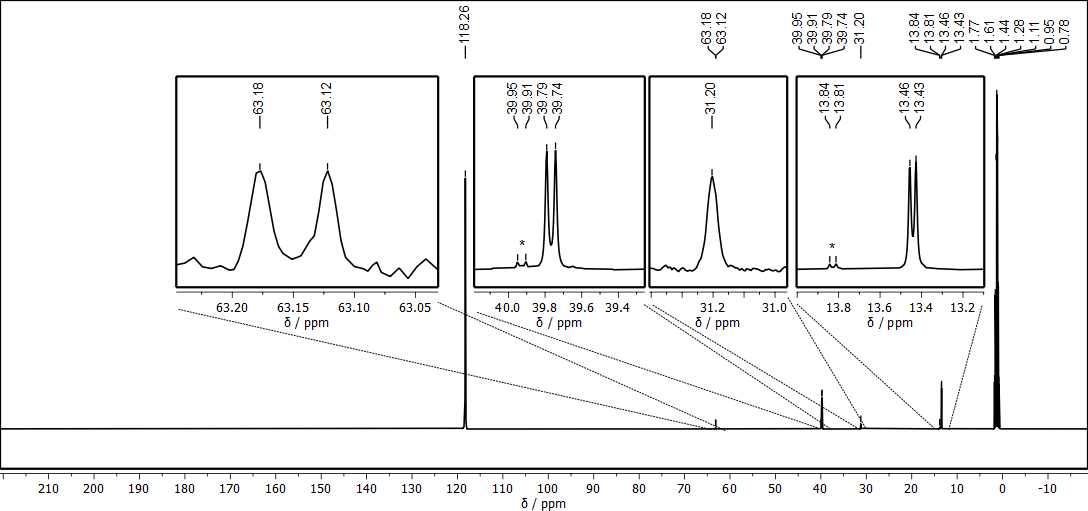


**Figure S7.** ^13^C{^1^H} NMR spectrum of [EtP_4_SCF_3_]Br (**2**) in CD_3_CN. Solvent signals: 1.32 ppm and 118.26 ppm. *[EtP_4_H] cation.


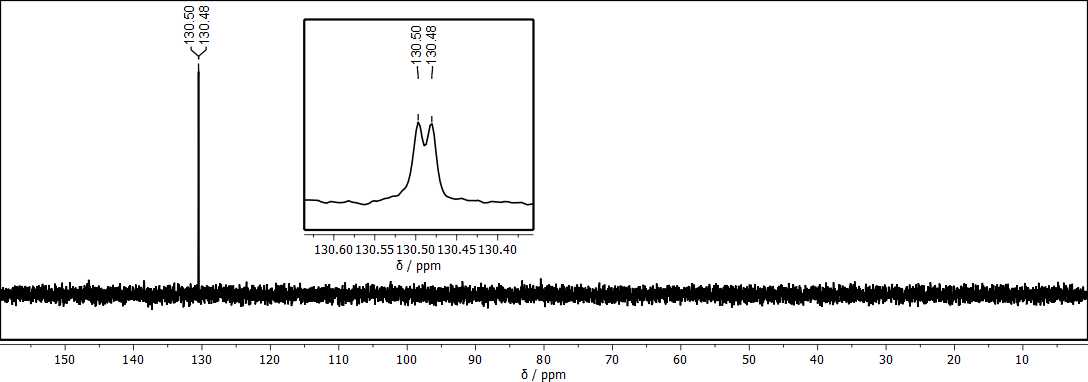


**Figure S8.** ^13^C{^19^F} NMR spectrum of [EtP_4_SCF_3_]Br (**2**) in CD_3_CN. O2P = −48.0 ppm.


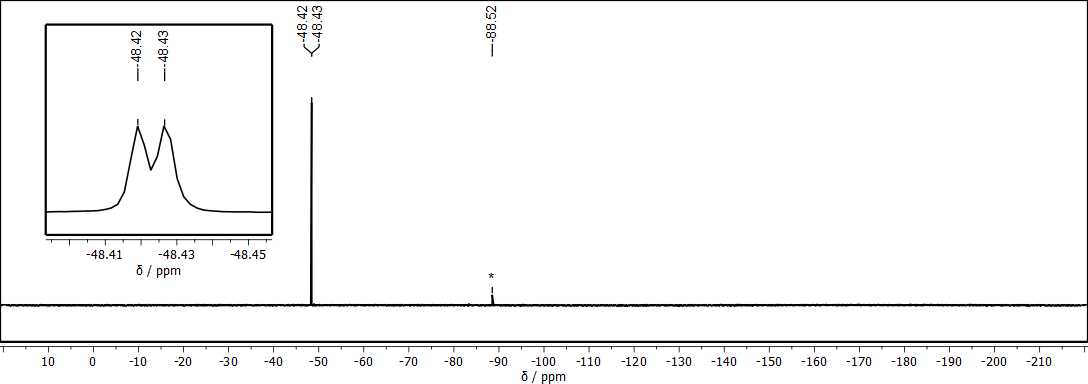


**Figure S9.** ^19^F NMR spectrum of [EtP_4_SCF_3_]Br (**2**) in CD_3_CN.


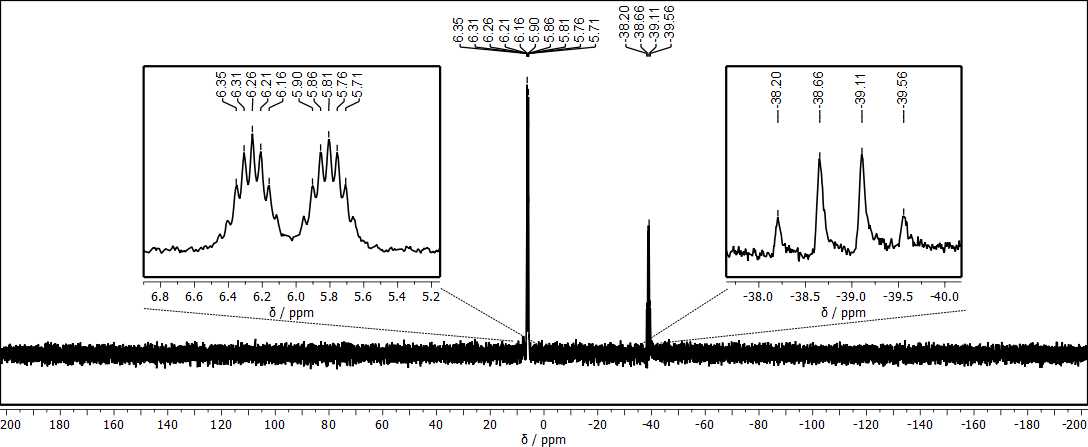


**Figure S10.** ^31^P NMR spectrum of [EtP_4_SCF_3_]Br (**2**) in CD_3_CN.

**4.3** NMR spectra of [EtP_4_SCF_3_]I (**3**)


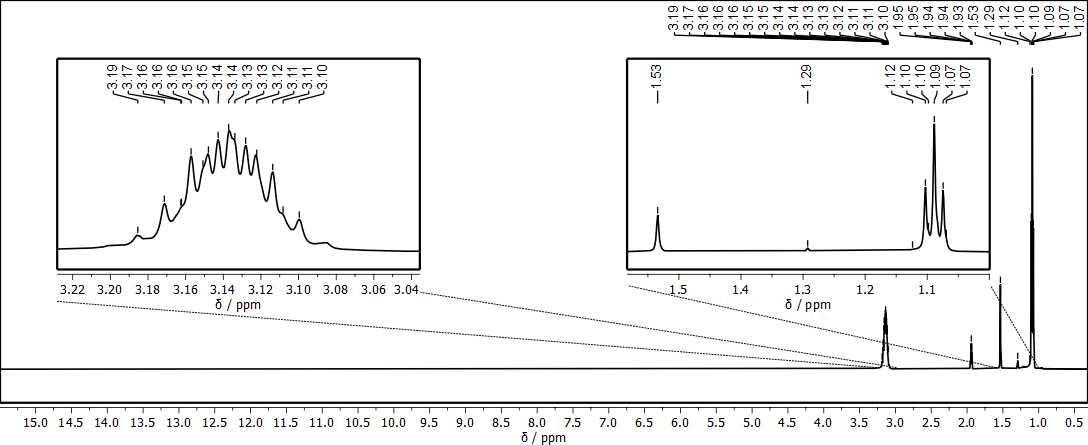


**Figure S11.** ^1^H NMR spectrum of [EtP_4_SCF_3_]I (**3**) in CD_3_CN. Solvent signal: 1.94 ppm.


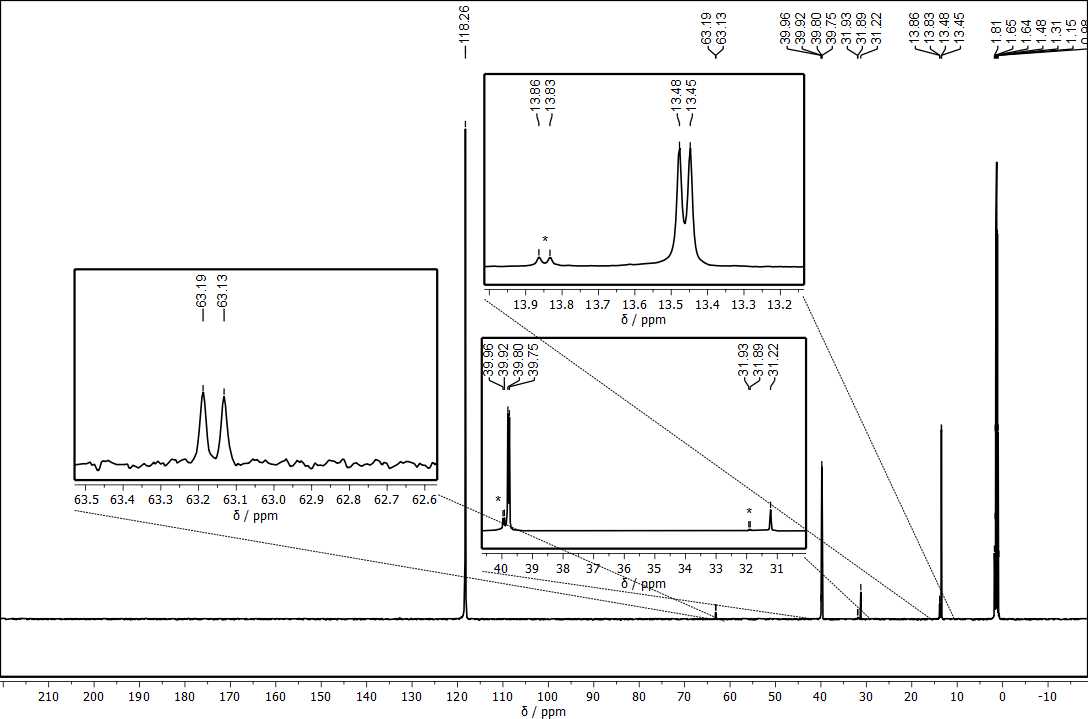


**Figure S12.** ^13^C{^1^H}NMR spectrum of [EtP_4_SCF_3_]I (**3**) in CD_3_CN. Solvent signals: 1.32 ppm and 118.26 ppm. *[EtP_4_H] cation.


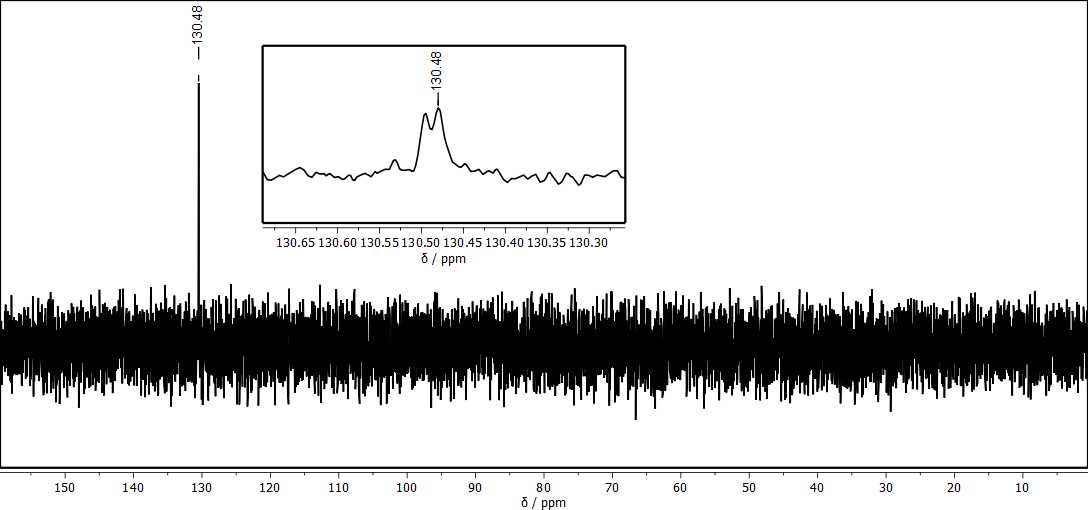


**Figure S13.** ^13^C{^19^F} NMR spectrum of [EtP_4_SCF_3_]I (**3**) in CD_3_CN. O2P = −48.0 ppm.


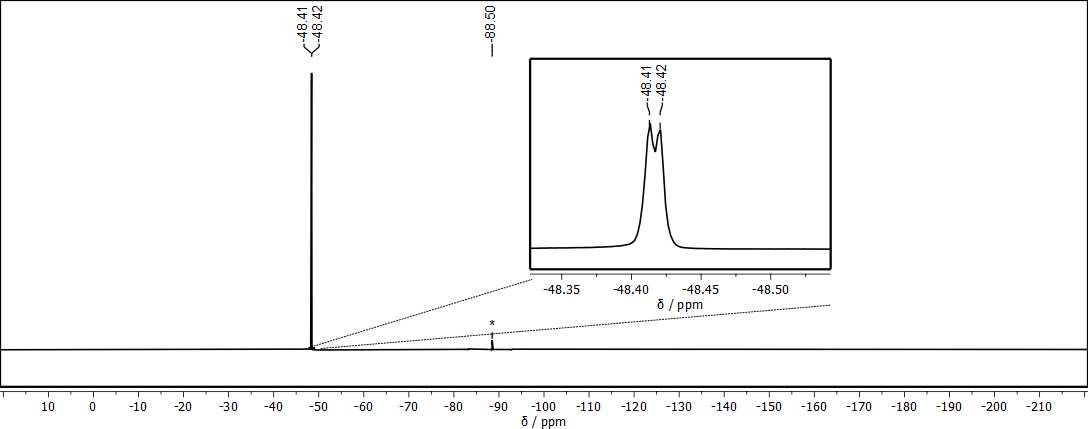


**Figure S14.** ^19^F NMR spectrum of [EtP_4_SCF_3_]I (**3**) in CD_3_CN.


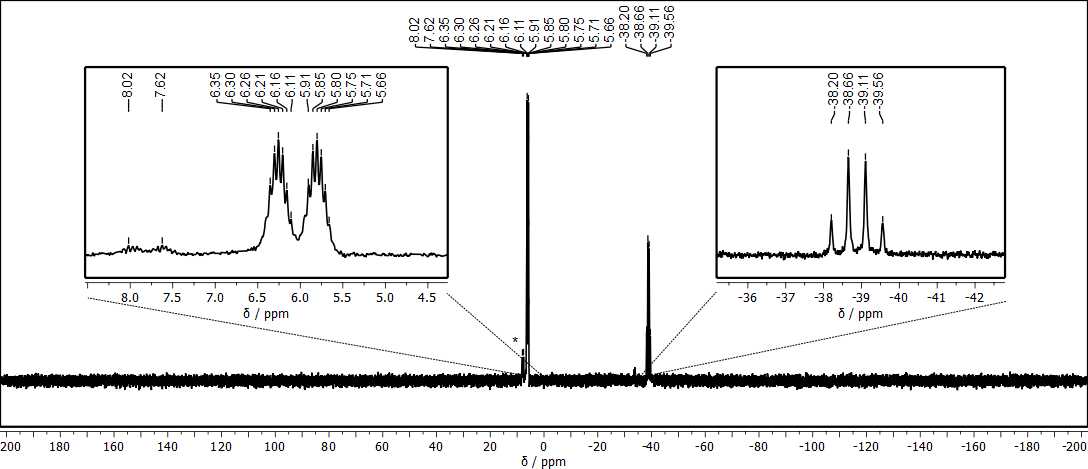


**Figure S15.** ^31^P NMR spectrum of [EtP_4_SCF_3_]I (**3**) in CD_3_CN. *[EtP_4_H] cation.

**4.4** NMR spectra of [({Et_2_N}_3_P=N)_3_PCl]Cl (**4**)


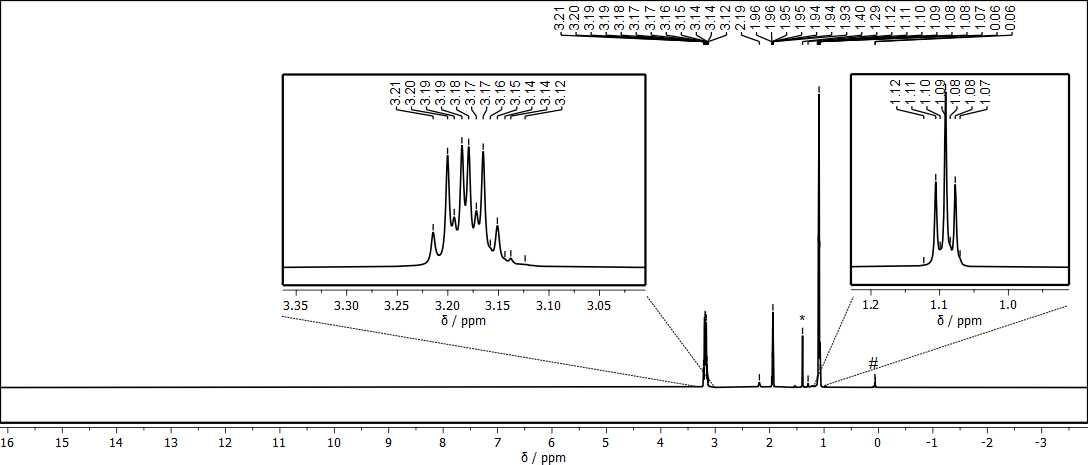


**Figure S16.** ^1^H NMR spectrum of [({Et_2_N}_3_P=N)_3_PCl]Cl (**4**) in CD_3_CN. Solvent signal: 1.94 ppm. *[H_3_N*^t^*Bu] cation, #silicon grease.


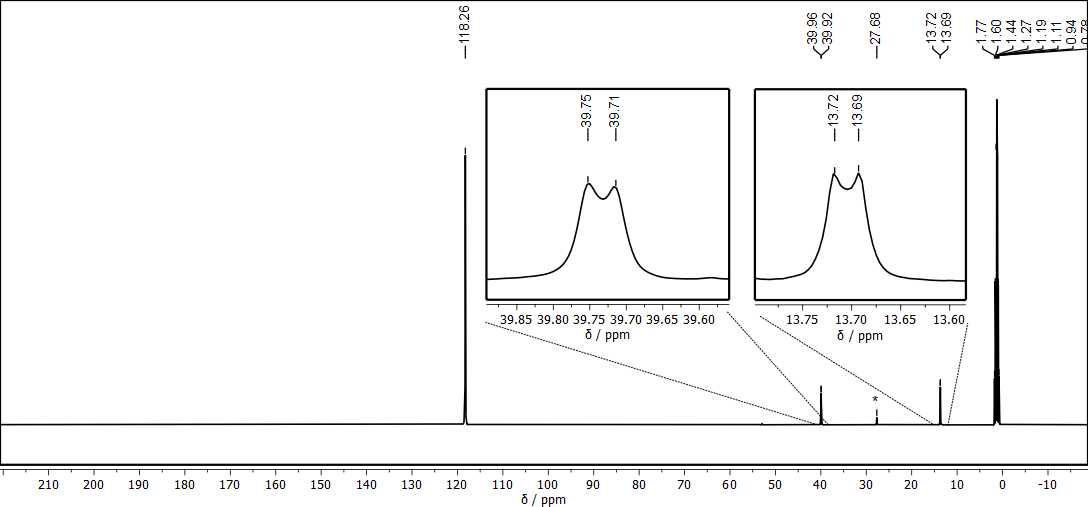


**Figure S17.** ^13^C{^1^H} NMR spectrum of [({Et_2_N}_3_P=N)_3_PCl]Cl (**4**) in CD_3_CN. Solvent signal: 1.32 ppm and 118.26 ppm. *[H_3_N^t^Bu] cation.


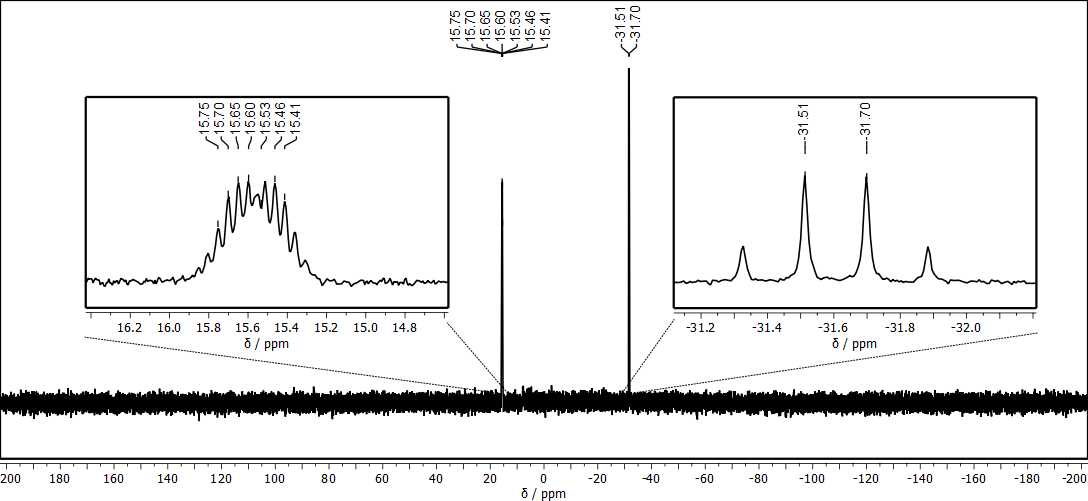


**Figure S18.** ^31^P NMR spectrum of [({Et_2_N}_3_P=N)_3_PCl]Cl (**4**) in CD_3_CN.

## **4.5** NMR spectrum of [({Et_2_N}_3_P=N)_3_PBr]Br (**5**)


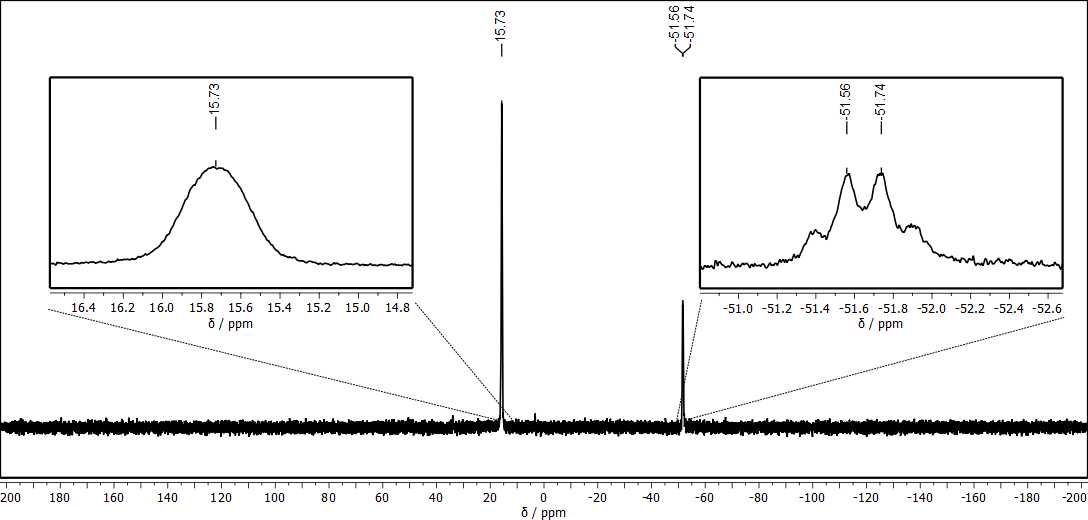


**Figure S19.** ^31^P NMR spectrum of [({Et_2_N}_3_P=N)_3_PBr]Br (**5**) in MeCN.

## **4.6** NMR spectrum from the reaction of **1** with Me_3_SiCl


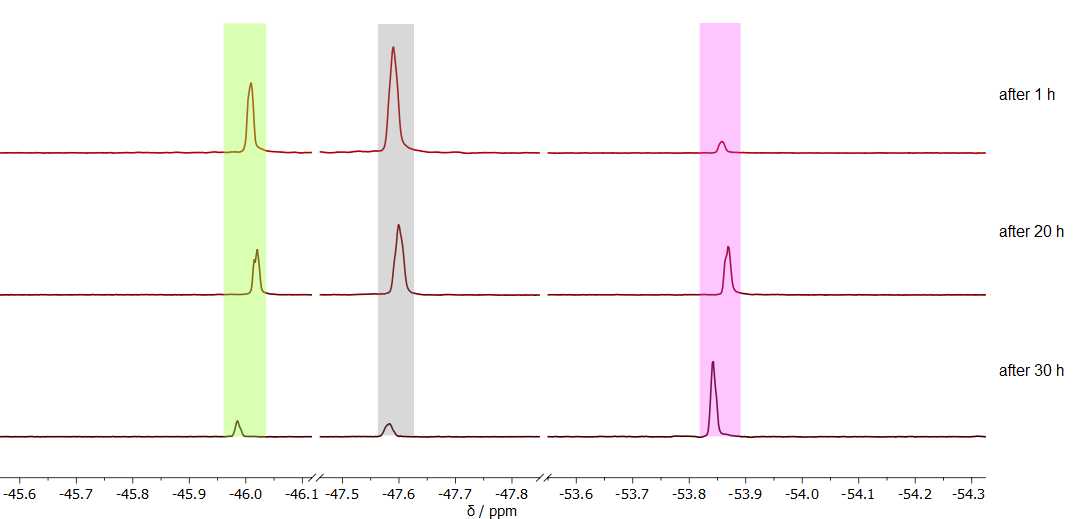


**Figure S20.** Section from the ^19^F NMR spectrum of the reaction of [EtP_4_SCF_3_][SCF_3_] (**1**) with Me_3_SiCl after the indicated reaction time. grey: [EtP_4_SCF_3_][SCF_3_], green: Me_3_SiN(SCF_3_)(^t^Bu), purple: HN(SCF_3_)(^t^Bu).

Monitoring the reaction *via* ^19^F NMR spectroscopy (Figure S20) reveals a new resonance at −45.9 ppm (green), shifted by 1.6 ppm relative to the resonance of the corresponding SCF_3_ moiety in **1** (−47.5 ppm, grey). This indicates a similar chemical environment and is consistent with the formation of Me_3_SiN(SCF_3_)(*^t^*Bu). Over the course of one day, an increase of the signal at −53.7 ppm (purple) is observed, which is attributed to HN(SCF_3_)(*^t^*Bu), presumably formed *via* hydrolysis of Me_3_SiN(SCF_3_)(*^t^*Bu). This is in line with the chemical shifts of previously reported SCF_3_ substituted amines.^[2,3]^

## **4.7** NMR spectra resulting from reactions of (F_3_CS)_2_ with iminophosphoranes

### **4.7.1** With (C_4_H_8_N)_3_P=N*^t^*Bu


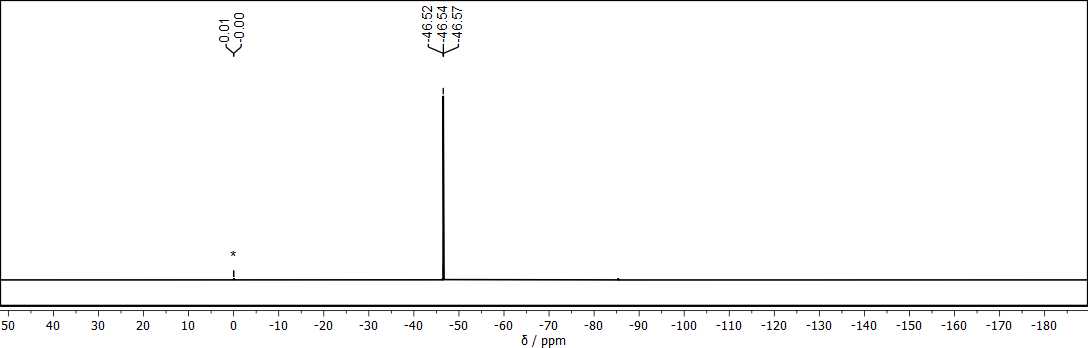


**Figure S21.** ^19^F NMR spectrum from the reaction of (C_4_H_8_N)_3_P=N^t^Bu and (F_3_CS)_2_ in n-pentane. O1P = −70 ppm. *CFCl_3_.


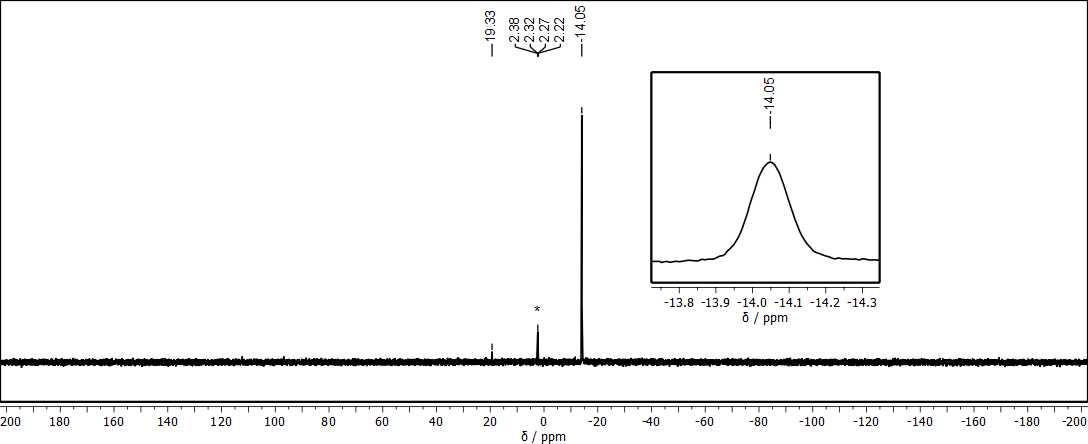


**Figure S22.** ^31^P NMR spectrum from the reaction of (C_4_H_8_N)_3_P=N^t^Bu and (F_3_CS)_2_ in n-pentane. *O=P(OMe)_3_.

### **4.7.2** With Ph_3_P=NSiMe_3_


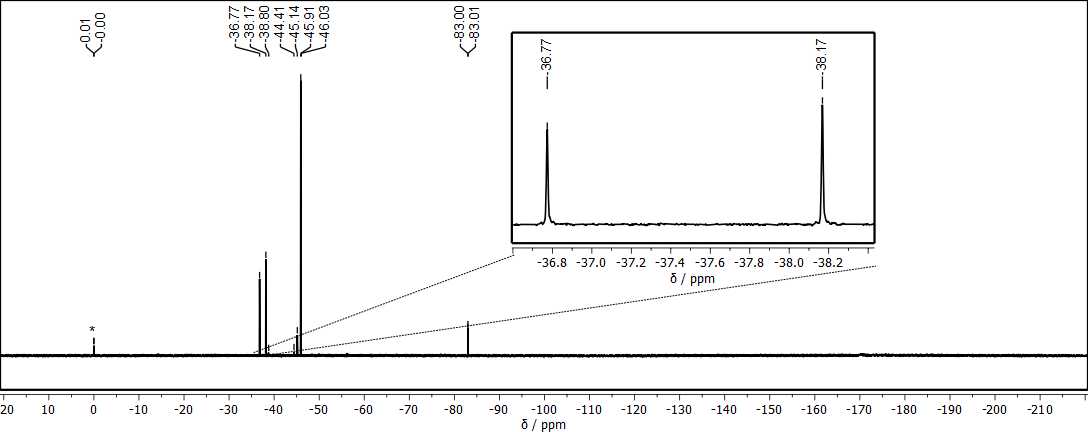


**Figure S23.** ^19^F NMR spectrum from the reaction of Ph_3_P=NSiMe_3_ and (F_3_CS)_2_ in MeCN. *CFCl_3_.


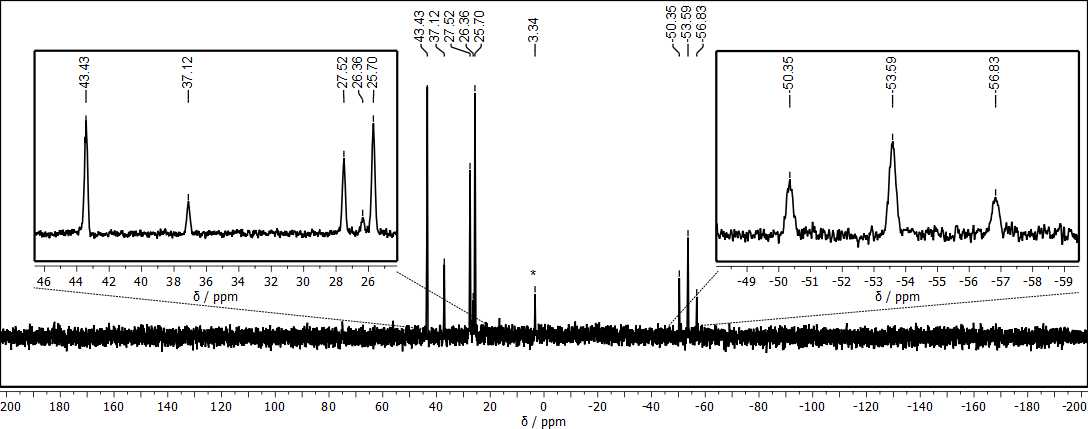


**Figure S24.** ^31^P NMR spectrum from the reaction of Ph_3_P=NSiMe_3_ and (F_3_CS)_2_ in MeCN. *O=P(OMe)_3_.

## **4.8** NMR spectra resulting from reactions of (F_3_CS)_2_ with phosphanes

### **4.8.1** With PMe_3_


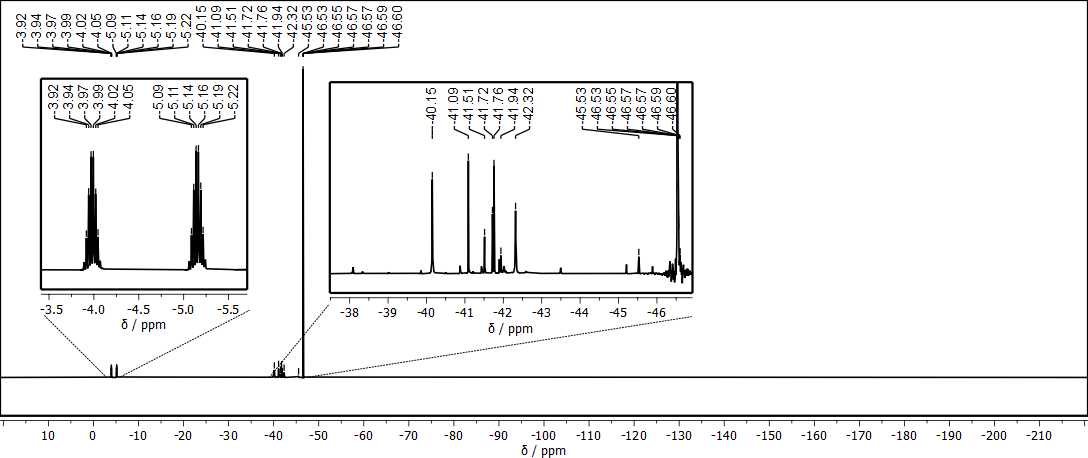


**Figure S25.** ^19^F NMR spectrum from the reaction of PMe_3_ and (F_3_CS)_2_ in Et_2_O.


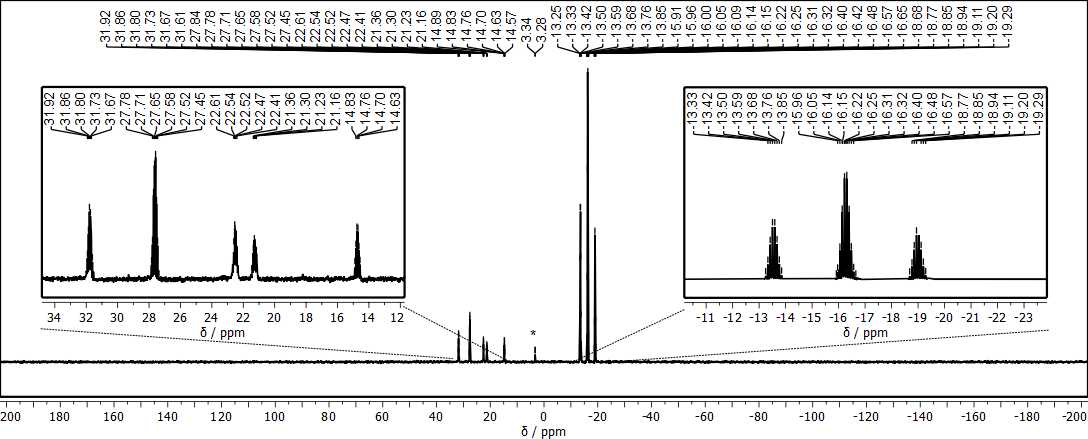


**Figure S26.** ^31^P NMR spectrum from the reaction of PMe_3_ and (F_3_CS)_2_ in Et_2_O. *O=P(OMe)_3_.

### **4.8.2** With P(NEt_2_)_3_


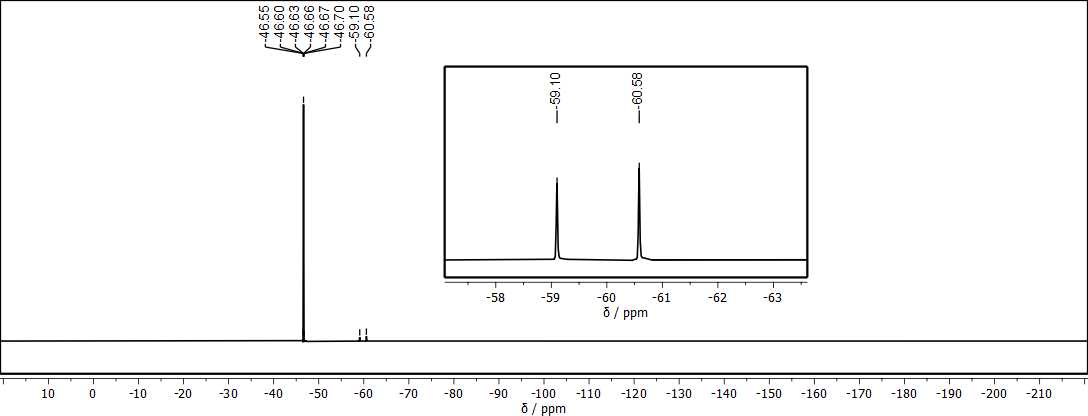


**Figure S27.** ^19^F NMR spectrum from the reaction of P(NEt_2_)_3_ and (F_3_CS)_2_ in Et_2_O.


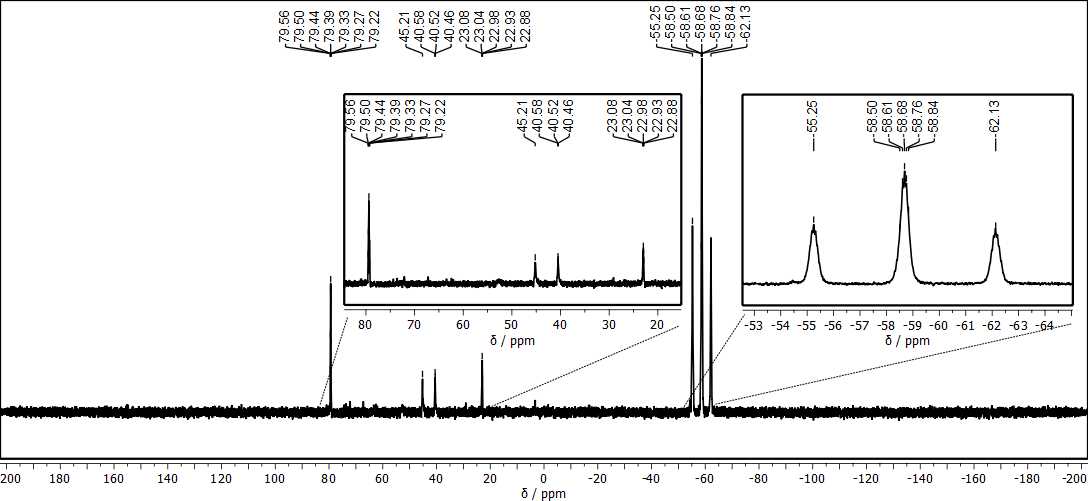


**Figure S28.** ^31^P NMR spectrum from the reaction of P(NEt_2_)_3_ and (F_3_CS)_2_ in Et_2_O.

**5 X-Ray diffraction data**

Single crystal X-ray diffraction analyses were performed on a Rigaku Supernova diffractometer using Mo-K*α* or Cu-K*α* radiation. Crystals were kept at 100.0(1) K during data collection. Using Olex2, the structure was solved with SHELXT structure solution program using intrinsic phasing and refined with olex2.refine refinement package using Least square minimisation.^[4−6]^ CCDC 2504752-2504756 contain the supplementary crystallographic data for this paper. These data can be obtained free of charge from the Cambridge Crystallographic Data Centre via <https://www.ccdc.cam.ac.uk/structures>.

In **1**: Disorder of the [SCF_3_]^−^ anion over three sites in a ratio of 72(S2A):20(S2B):8(S2C) (Figure S29). Due to low occupancy, the third part was restrained to be the same as the part with the highest occupancy. Displacement parameters of disordered atoms were also restraint.

**Figure S29** Disorder of the [SCF_3_]^−^ anion in the X-ray crystal structure of [EtP4SCF3][SCF3] (**1**), where thermal ellipsoids are shown at the 50% probability level. The cation was omitted for clarity.

In **2**: Disorder of one CHCl_3_ molecule over two sites in a ratio of 73:28, restraints were used inside this disorder. An additional, partly occupied highly disordered CHCl_3_ molecule could not be modeled reasonably, therefore, a solvent mask was calculated and 202 electrons were found in a volume of 662 A^3^ in 2 voids per unit cell. This is consistent with the presence of 0.9 CHCl_3_ per asymmetric unit which account for 209 electrons per unit cell.

In **3**: Disorder of one CHCl_3_ over two sites in a ratio of 81:19, restraints were used inside this disorder.

In **4**: Disorder of C6 over two sites in a ratio of 65:35. Disorder of Cl2 and Cl3 over two sites in a ratio of 85(Cl2A, Cl3A):15(Cl2B, Cl3B), restraints were used inside the disordered parts. A residual electron density in the solvent area was modeled as 9% solvent water molecule per formula unit (Figure S30).

**Figure S30** Disorder of both Cl^−^ anions in the X-ray crystal structure of [({Et_2_N}_3_P=N)_3_PCl]Cl$\cdot$[H_3_N^t^Bu]Cl where thermal ellipsoids are shown at the 50% probability level. The second cation was omitted for clarity.

In **5**: Disorder of C28 over two sites in a ratio of 53:47. All hydrogen atoms, except the disordered ones, were refined isotropically.

**Table S1.** X-Ray diffraction data of compounds **1**-**3**.

|  | [EtP_4_SCF_3_][SCF_3_] | [EtP_4_SCF_3_]Br | [EtP_4_SCF_3_]I $\cdot$ 4 CHCl_3_ |
| --- | --- | --- | --- |
| Empirical Formula | C_42_H_99_F_6_N_13_P_4_S_2_ | C_42.9_H_100.9_BrCl_5.7_F_3_N_13_P_4_S | C_45_H_103_Cl_12_F_3_IN_13_P_4_S |
| Formula weight /g$\cdot$mol^−1^ | 1088.34 | 1293.98 | 1591.64 |
| Temperature /K meas. | 100.0(1) | 100.0(1) | 100.0(1) |
| Crystal system | monoclinic | monoclinic | orthorhombic |
| Space group | *P*2_1_/*c* | *P*2_1_/*n* | *Pbca* |
| *a*/Å | 19.2320(2) | 12.07580(10) | 22.3020(2) |
| *b*/Å | 11.94941(13) | 14.3428(2) | 25.03886(19) |
| *c*/Å | 24.9139(3) | 37.1170(4) | 26.37249(19) |
| *β*/° | 94.0107(11) | 92.4660(10) | 90 |
| Volume/Å^3^ | 5711.47(12) | 6422.74(13) | 14726.9(2) |
| *Z* | 4 | 4 | 8 |
| *ρ*_calc_/g$\cdot$cm^−3^ | 1.266 | 1.338 | 1.436 |
| *μ*/mm^−1^ | 2.419 | 1.059 | 8.905 |
| *F*(000) | 2352.0 | 2737.0 | 6592.0 |
| Crystal size /mm^3^ | 0.18 × 0.09 × 0.04 | 0.31 × 0.2 × 0.06 | 0.34 × 0.17 × 0.03 |
| Radiation /Å | Cu K*α*  ($\lambda$ = 1.54184) | Mo K*α*  ($\lambda$ = 0.71073) | Cu K*α*  ($\lambda$ = 1.54184) |
| 2*Θ* range for data collection/° | 4.606 to 152.508 | 4.35 to 63.012 | 6.276 to 152.622 |
| Index ranges | -24 ≤ *h* ≤ 24,  -15 ≤ *k* ≤ 15,  -30 ≤ *l* ≤ 31 | -17 ≤ *h* ≤ 17,  -21 ≤ *k* ≤ 21,  -54 ≤ *l* ≤ 54 | -27 ≤ *h* ≤ 25,  -31 ≤ *k* ≤ 31,  -33 ≤ *l* ≤ 33 |
| Reflections collected | 102631 | 187773 | 272518 |
| Independent  reflections | 11863 [*R*_int_ = 0.0484, *R*_sigma_ = 0.0237] | 21400 [*R*_int_ = 0.0751, *R*_sigma_ = 0.0449] | 15272 [*R*_int_ = 0.0951, *R*_sigma_ = 0.0307] |
| Reflections with *I>=2u(I)* | 10575 | 16786 | 13416 |
| Data/restraints/parameters | 11863/404/712 | 21400/87/653 | 15272/99/761 |
| Goodness-of-fit on *F*^2^ | 1.021 | 1.029 | 1.038 |
| Final *R* indexes [*I>=*2*u(I)*] | *R*_1_ = 0.0391,  w*R*_2_ = 0.1021 | *R*_1_ = 0.0528, w*R*_2_ = 0.1140 | *R*_1_ = 0.0421, w*R*_2_ = 0.1110 |
| Final *R* indexes [all data] | *R*_1_ = 0.0443,  w*R*_2_ = 0.1061 | *R*_1_ = 0.0715, w*R*_2_ = 0.1213 | *R*_1_ = 0.0487, w*R*_2_ = 0.1171 |
| Max. residuals/e$\cdot$Å^−3^ | 0.69/−0.51 | 0.75/−1.14 | 0.97/−1.22 |
| CCDC | 2504752 | 2504753 | 2504754 |

**Table S2.** X-Ray diffraction data of compounds **4** and **5**.

|  | [({Et_2_N}_3_P=N)_3_PCl]Cl$\cdot$[H_3_N*^t^*Bu]Cl | [({Et_2_N}_3_P=N)_3_PBr]Br$\cdot$[H_3_N*^t^*Bu]Br |
| --- | --- | --- |
| Empirical Formula | C_40_H_102.19_Cl_3_N_13_O_0.09_P_4_ | C_40_H_102_Br_3_N_13_P_4_ |
| Formula weight /g∙mol^−1^ | 997.20 | 1128.95 |
| Temperature /K meas. | 100.0(1) | 100.0(1) |
| Crystal system | triclinic | triclinic |
| Space group | *P*$\bar{1}$ | *P*$\bar{1}$ |
| *a*/Å | 10.57130(10) | 10.4204(3) |
| *b*/Å | 14.4228(2) | 14.7554(6) |
| *c*/Å | 20.1894(2) | 20.4246(7) |
| *α*/° | 110.0590(10) | 110.542(3) |
| *β*/° | 101.9720(10) | 102.114(3) |
| *γ*/° | 90.8930(10) | 90.529(3) |
| Volume/Å^3^ | 2815.98(6) | 2863.45(18) |
| *Z* | 2 | 2 |
| *ρ*_calc_/g$\cdot$cm^−3^ | 1.176 | 1.309 |
| *μ*/mm^−1^ | 2.854 | 2.264 |
| *F*(000) | 1090.0 | 1196.0 |
| Crystal size /mm^3^ | 0.2 × 0.1 × 0.08 | 0.24 × 0.13 × 0.03 |
| Radiation /Å | Cu K*α*  (*λ* = 1.54184) | Mo K*α*  (*λ* = 0.71073) |
| 2*Θ* range for data collection/° | 4.784 to 152.168 | 5.2 to 65.664 |
| Index ranges | -13 ≤ *h* ≤ 13,  -17 ≤ *k* ≤ 18,  -25 ≤ *l* ≤ 25 | -15 ≤ *h* ≤ 15, -22 ≤ *k* ≤ 20,  -31 ≤ *l* ≤ 31 |
| Reflections collected | 74549 | 64799 |
| Independent  reflections | 11653 [*R*_int_ = 0.0379, *R*_sigma_ = 0.0225] | 19211 [*R*_int_ = 0.0424, *R*_sigma_ = 0.0515] |
| Reflections with *I>=*2*u(I)* | 10507 | 14678 |
| Data/restraints/parameters | 11653/37/604 | 19211/1/941 |
| Goodness-of-fit on *F*^2^ | 1.027 | 1.048 |
| Final R indexes [ *I>=*2*u(I)*] | *R*_1_ = 0.0302,  w*R*_2_ = 0.0787 | *R*_1_ = 0.0378,  w*R*_2_ = 0.0773 |
| Final *R* indexes [all data] | *R*_1_ = 0.0344,  w*R*_2_ = 0.0819 | *R*_1_ = 0.0620,  w*R*_2_ = 0.0857 |
| Max. residuals/e$\cdot$Å^−3^ | 0.43/−0.49 | 0.81/−0.75 |
| CCDC | 2504755 | 2504756 |

**6 Solid-state structures of compounds 2 and 3**

**Figure S31.** Molecular structure of [EtP_4_SCF_3_]Br (**2**). Disorder of one CHCl_3_ molecule over two sites in ratio of 73:28. Minor occupied atoms and hydrogen atoms of the cation are omitted for clarity. Thermal ellipsoids are shown at the 50% probability level. Selected bond lengths / pm and angles / °: P1-N1 175.8(2), N1-C1 153.7(2), N1-S1 168.4(2), S1-C41 180.8(2), C41-F3 133.3(2), P1-N1-S1 116.5(1), P1-N1-C1 123.0(1), N1-S1-C41 104.9(1), S1-C41-F3 114.1(1).

**Figure S32.** Molecular structure of [EtP_4_SCF_3_]I (**3**)$\cdot$4 CHCl_3_. Hydrogen atoms of the cation are omitted for clarity. Thermal ellipsoids are shown at the 50% probability level. Selected bond lengths / pm and angles / °: P1-N1 175.3(2), N1-C1 153.1(3), N1-S1 168.5(2), S1-C41 180.6(3), C41-F3 132.8(3), P1-N1-S1 116.4(1), P1-N1-C1 122.7(2), N1-S1-C41 103.5(1), S1-C41-F3 114.0(2).

**7 References**

[1] R. F. Weitkamp, B. Neumann, H.-G. Stammler, B. Hoge, *Angew. Chem, Int. Ed.* **2019**, *58*, 14633–14638.

[2] A. Haas, M. Häberlein, C. Krüger, *Chem. Ber.* **1976**, *109*, 1769–1778.

[3] A. Ferry, T. Billard, B. R. Langlois, E. Bacqué, *J. Org. Chem.* **2008**, *73*, 9362–9365.

[4] L. J. Bourhis, O. V. Dolomanov, R. J. Gildea, J. A. K. Howard, H. Puschmann, *Acta Crystallogr, Sect. A: Found. Adv.* **2015**, *71*, 59–75.

[5] G. M. Sheldrick, *Acta Crystallogr, Sect. C: Struct. Chem.* **2015**, *71*, 3–8.

[6] O. V. Dolomanov, L. J. Bourhis, R. J. Gildea, J. A. K. Howard, H. Puschmann, *J. Appl. Crystallogr.* **2009**, *42*, 339–341.
